# Functional Characterization of BoaMYB51s as Central Regulators of Indole Glucosinolate Biosynthesis in *Brassica oleracea* var. *alboglabra* Bailey

**DOI:** 10.3389/fpls.2018.01599

**Published:** 2018-11-06

**Authors:** Congxi Cai, Wenxin Yuan, Huiying Miao, Mingdan Deng, Mengyu Wang, Jiayao Lin, Wei Zeng, Qiaomei Wang

**Affiliations:** ^1^State Agriculture Ministry Laboratory of Horticultural Crop Growth and Development, Hangzhou, China; ^2^Zhejiang Provincial Key Laboratory of Horticultural Plant Integrative Biology, Hangzhou, China; ^3^School of Horticulture and Plant Protection, Yangzhou University, Yangzhou, China

**Keywords:** BoaMYB51, Chinese kale, indole glucosinolate, protein-protein interaction, transcription regulation

## Abstract

R2R3-MYB transcription factor MYB51 is known to control indole glucosinolate (indole GSL) biosynthesis in *Arabidopsis*. Here, two copies of *BoaMYB51* have been isolated in Chinese kale (*Brassica oleracea* var. *alboglabra* Bailey), designated *BoaMYB51.1* and *BoaMYB51.2*, which exhibit overlapping but distinct expression levels among different organs and respond to signaling molecules in a similar pattern. It has been demonstrated a structural and functional conservation between BoaMYB51s and AtMYB51 by phylogenetic analysis, complementation studies and transient expression assay. To further investigate the transcriptional mechanism, we identified the transcriptional activation domain (TAD) and putative interacting proteins of BoaMYB51s by means of yeast (*Saccharomyces cerevisiae*) two hybrid. Using tobacco (*Nicotiana benthamiana*) transient expression assay, we confirmed that the carboxy-end is required for transcriptional activation activity of BoaMYB51s. In addition, several BoaMYB51-interacting proteins have been identified by yeast two-hybrid screening. These results provide important insights into the molecular mechanisms by which MYB51 transcriptionally regulates indole GSL biosynthesis.

## Introduction

Chinese kale (*Brassica oleracea* var. *alboglabra* Bailey), a biennial vegetable belonging to the family of *Brassicaceae*, is widespread in southern China and Southeast Asia, with a large growing area and a marketable supply in these regions. Generally, Chinese kale is consumed for its bolting stems as common edible parts, and the tender rosette leaves and sprouts are also widely consumed (Wang et al., 2017). In addition to good flavor, numerous studies indicate that Chinese kale has abundant glucosinolates (GSLs) (Sun et al., 2011; Qian et al., 2016).

GSLs are a class of nitrogen- and sulfur-containing amino acid-derived secondary metabolites, which are common in members of the *Brassicaceae* family including the model plant *Arabidopsis thaliana* and agriculturally important *Brassica* crops such as Chinese cabbage (*Brassica rapa* ssp. *pekinensis*), broccoli (*Brassica oleracea* var. *italica*), and cauliflower (*Brassica oleracea* var. *botrytis*) (Halkier and Gershenzon, 2006; Mithen et al., 2010; Cai et al., 2016; Seo et al., 2016; Capriotti et al., 2018). In recent years, GSL have evolved as a model system for study of secondary metabolite in plants. Depending on the origin of the core amino acid, GSLs are generally classified into three groups: aliphatic GSL derived from methionine, leucine, isoleucine or valine; indole GSL derived from tryptophan; and benzolic GSL derived from tyrosine or phenylalanine (Fahey et al., 2001; Kliebenstein et al., 2001; Wittstock and Halkier, 2002), of which more than 200 different GSLs have been identified (Clarke, 2010). It is known that GSLs and their breakdown products contribute to the protective effects against cancer (Gross et al., 2000; Mithen et al., 2003; Grubb and Abel, 2006). Besides the benefits to the human health, these compounds also play an active role in plant defense against microbial pathogens (Clay et al., 2009) as well as herbivorous insects (Kliebenstein et al., 2002; Levy et al., 2005), and therefore, it will be a potential strategy for improvement of *Brassica* vegetables by regulating GSL.

Over the past two decades, indole GSL biosynthetic pathway has been well elucidated, with the almost complete characterization of enzymes involved (Halkier and Gershenzon, 2006). The composition and content of GSLs vary drastically in response to environmental stimuli, and GSL biosynthesis can be controlled by multiple signals. An understudied aspect of GSL research is to illustrate this regulatory machinery. Recent observations have begun to provide evidence that transcriptional regulation plays a central role in biosynthesis of secondary metabolites (Stracke et al., 2007; Gonzalez et al., 2008). R2R3-MYB transcription factors have been shown to function in a variety of plant-specific processes, which control development, primary and secondary metabolism and response to biotic and abiotic stresses (Dubos et al., 2010). In *A. thaliana*, six R2R3-MYB transcription factors having a characteristic “[L/F]LN[K/R]VA” motif (Stracke et al., 2001) are known to be involved in transcriptional regulation of aliphatic GSL (MYB28, MYB29, and MYB76), and indole GSL (MYB34, MYB51, and MYB122) (Gigolashvili et al., 2007a,b, 2008; Malitsky et al., 2008; Sønderby et al., 2010). Among them, MYB51 controls indole GSL biosynthesis mainly in the shoots and have been demonstrated to exclusively trans-activates the expression of indole GSL biosynthetic genes and differentially respond to phytohormone signaling molecules such as abscisic acid (ABA), jasmonate (JA), ethylene (ETH), and salicylate (SA) (Gigolashvili et al., 2007a; Frerigmann and Gigolashvili, 2014), but the underlying transcriptional mechanism remains relatively poorly defined.

A class of JA-signaled bHLH transcription factors (MYC2, MYC3, and MYC4) has been shown to interact with the six GSL-related MYB transcription factors, respectively (Schweizer et al., 2013; Frerigmann et al., 2014). These MYB-bHLH protein complexes have been linked to GSL biosynthesis regulating, and similar scenarios can also be found in other plant secondary metabolites (Gonzalez et al., 2008; Xu et al., 2014). Since previous analyses of the reference genome sequence of *B. oleracea* identified a whole-genome triplication (WGT) event after its divergence from a common ancestor of *A. thaliana* in the *Brassica* ancestor, most genes in Chinese kale were found to be duplicated or triplicated (Cheng et al., 2014). Thus, construction of Chinese kale cDNA prey library for yeast two-hybrid screening will be enriched for cDNAs of naturally co-occurring proteins, which will increase the possibility to identify novel GSL-related MYB interactors and enable us to seek other types of MYB-bHLH protein complex regulating GSL biosynthesis.

Despite the identification of aliphatic GSL biosynthesis regulator MYB28 and MYB29 in *Brassica rapa ssp. pekinensis* (Kim et al., 2013), *B. juncea* (Augustine et al., 2013), and *B. oleracea* (Araki et al., 2013; Yin et al., 2017), none of the transcription factors regulating indole GSL in *Brassica* crops have been well investigated to date. Previous study only identified one BoMYB51 from broccoli (Yu et al., 2018). Here, we report that both two *BoaMYB51* genes have been isolated with identification of their transcriptional activation domain (TAD) and novel protein interactors. Our results highlight the importance of BoaMYB51s in regulating indole GSL biosynthesis in Chinese kale, which could be potential in improvement of pest and pathogen resistance as well as health benefits of *B. oleracea* by metabolic engineering.

## Materials and methods

### Plant material and cultivation conditions

Chinese kale cultivar “DSCH” germinated and grown in Zhejiang University (Hangzhou, China) was used as material in this study. Chinese kale seeds were sterilized for 30 s in 75% ethanol and washed with sterile water twice, and then immersed in 10% bleach for 2 min, followed by washing with sterile water five times. They were then soaked in sterile-distilled water for 48 h at 25°C in dark. Chinese kale sprouts were cultured in petri dishes with three pieces of wet filter paper in a plant growth chamber at 25°C with a 16-h-light/8-h-dark photoperiod. Five-day-old sprouts and six-month-old Chinese kale plants were used to collect different organs to analyze *BoaMYB51* expression patterns. For epi-brassinolide (eBL) (Sigma), methyl-jasmonate (MeJA) (Sigma), SA (Sigma), and flagelin22 (flg22) (Chinese Peptide, Hangzhou, China) treatment, 5-day-old Chinese kale sprouts were grown in sterile half-strength Murashige and Skoog (MS) media at 25°C with a 16-h-light/8-h-dark photoperiod.

*Arabidopsis* mutant *myb34myb51* (Frerigmann and Gigolashvili, 2014) was used for functional complementation studies. *Arabidopsis* seeds were sterilized for 12 min in 10% bleach and washed with sterile water 5 times, and then were stratified for 3 days at 4°C. *Arabidopsis* plants were grown in sterile half-strength Murashige and Skoog (MS) media at 22°C with a 16-h-light/8-h-dark photoperiod.

### Phylogenetic analysis

Multiple protein sequence alignments were performed using Clustal W. The phylogenetic tree was produced based on the amino acid sequences of MYB34, MYB51, and MYB122 proteins from *B. rapa, B. oleraceae*, and *A. thaliana* by applying the NJ (Neighbor-joining) method with MEGA v.7.0 software (Kumar et al., 2016), and bootstrap values with 1,000 replicates were calculated.

### RNA extraction and qRT-PCR analysis

Plant samples were ground using Tissuelyser-24 (Jingxin, Shanghai, China). Total RNA was isolated as described previously with minor modifications (Guo et al., 2013). Reverse transcription was performed using the PrimeScript™ RT reagent Kit with gDNA Eraser (Takara). qRT-PCR was performed on ABI StepOne Real-time PCR System (Thermo Fisher). *BoaACTIN2* gene was used as an endogenous control and the expression of other genes was computed using the 2^−ΔΔ*CT*^ method. Primers used are listed in Supplementary Table 1. Data were analyzed from three independent sets of biological replicates.

### DNA constructs and plant transformation

Constructs for plant transformation were generated using Gateway system (Invitrogen). The full-length coding DNA sequences (CDS) of *BoaMYB51*.1 and *BoaMYB51.2* were amplified with Gateway-compatible primers (see Supplementary Table 2). The PCR product was cloned using pENTR/D-TOPO CLONING KIT (Invitrogen) and then recombined with the binary vector pGWB2 (Nakagawa et al., 2007) to generate the *35S*_*pro*_*:BoaMYB51.1* and *35S*_*pro*_*:BoaMYB51.2* constructs. For the generation of *BoaMYB51*_*pro*_*:GUS* constructs, the promoter regions (~2 kb) of the two *BoaMYB51* genes were isolated from genomic DNA of and cloned into the gateway binary vector pGWB3 (Nakagawa et al., 2007). Histochemical analysis was performed as previously described (Sun et al., 2009). Primers are tabulated in Supplementary Table 2. The above constructs were then transformed into *Agrobacterium tumefaciens* strain GV3101, which was used for transformation of *Arabidopsis* plants by floral dip method.

### GSL assay

GSL were extracted and analyzed as previously described (Guo et al., 2013). Extract was applied to a DEAE-Sephadex A-25 (35 mg) column (Pyridine acetate form) (Sigma). ONPG (Sigma) was used as an internal standard for HPLC analysis. The GSL concentration was expressed as μmol g^−1^fresh weight (FW).

### Transient expression assay

The transient expression assays were carried out in tobacco leaves as previously described (Zhai et al., 2015). The promoters of *BoaCYP79B2.1, BoaCYP83B1, BoaSOT16.1, BoaCYP79F1*, and *AtCYP83B1* were amplified and cloned into entry vector using the pENTR/D-TOPO CLONING KIT (Thermo Fisher). Then promoters were fused with the luciferase reporter gene *LUC* into pGWB35 and pGreenII 0800-LUC to generate the reporter constructs *BoaCYP79B2.1*_*pro*_*:LUC, BoaCYP83B1*_*pro*_*:LUC, BoaSOT16.1*_*pro*_*:LUC, BoaCYP79F1*_*pro*_*:LUC*, and *AtCYP83B1*_*pro*_*:LUC*. Effector constructs *35S*_*pro*_*:BoaMYB51.1-YFP, 35S*_*pro*_*:BoaMYB51.2-YFP*, and *35S*_*pro*_*:BoaMYB28.1-YFP* were generated using ClonExpress II One Step Cloning Kit (Vazyme Biotech). We used a low-light cooled CCD imaging apparatus (NightOWL II LB983) to capture the LUC image and to count luminescence intensity. The leaves were sprayed with 100 mM luciferin (Promega) and were placed in darkness for 3 min before luminescence detection.

### Transactivation activity assay in yeast

Full-length CDS of *BoaMYB51.1* and *BoaMYB51.2* and their derivatives were amplified with listed primers (see Supplementary Table 2). PCR products were recombined with the pGBKT7 vector using ClonExpress II One Step Cloning Kit (Vazyme Biotech) for fusion with the BD domain at their N terminal. The resulting constructs were then transformed into the yeast strain *Saccharomyces cerevisiae* AH109, and the presence of the transgenes was verified by PCR and growth on an SD/-Trp plate. The Matchmaker GAL4 two-hybrid systems (Clontech) was used for the transactivation activity assay. Each yeast liquid culture was serially diluted to OD_600_ = 1.0, and 5 μl of each dilution was spread on the plates containing SD/-Ade/-His/-Trp synthetic dropout medium.

### Y2H assays

The TAD deletion constructs of BoaMYB51.1 and BoaMYB51.2 were used for the Y2H screening of the pGADT7-based Chinese kale cDNA library, which was generated with mRNAs isolated from Chinese kale sprouts, leaves, roots, stems and flowers. Y2H assays were based on Matchmaker GAL4 two-hybrid systems (Clontech). Yeast transformants were exhaustively selected on SD/-Ade/-His/-Leu/-Trp/X-α-Gal medium. Putative BoaMYB51 interacting clones were characterized and sequenced.

### Statistical analysis

Statistical analysis was performed using the SPSS package program version 11.5 (SPSS inc. Chicago, IL, USA). For Figure 7, Data was analyzed by one-way ANOVA, followed by Turkey's HSD multiple comparison test. The values are reported as means with their standard error for all results. Differences were considered significant at *p* < 0.05.

### Accession numbers

Chinese kale sequence information can be found in the *Brassica* database (BRAD, http://brassicadb.org) under the following accession numbers: *BoaMYB51.1* (Bol013207), *BoaMYB51.2* (Bol030761), *BoaMYB28.1* (Bol017019), *BoaCYP79B2.1* (Bol018585), *BoaCYP83B1* (Bol033477), *BoaSOT16.1* (Bol026200), *BoaCYP79F1* (Bol038222), *BoaBIM1.1* (Bol043819), *BoaBIM1.2* (Bol008832), and *BoaACTIN2* (Bol030974). All *Arabidopsis* genes used in this article are referenced in the Arabidopsis Genome Initiative under the following accession numbers: *AtMYB51* (At1G18570), *AtCYP83B1* (At4G31500) and *AtSOT16* (At1G74100).

## Results

### Isolation of the two BoaMYB51 homologs from Chinese kale

According to *Brassica* database (BRAD, http://brassicadb.org), in *B. oleracea* genome, there are two *MYB51* homologs distributed in different chromosomes, namely *BoaMYB51.1* (Bol013207, C08) and *BoaMYB51.2* (Bol030761, C05). First, primers based on reference sequences from BRAD were used to isolate the two *BoaMYB51* genes from Chinese kale cultivar “DSCH.” Next, the full-length coding sequences of two *BoaMYB51* genes were confirmed with multiple amplifications in other Chinese kale cultivars. As a result, the length of CDS of *BoaMYB51.1* is 1002 bp encoding a protein of 333 aa, whereas the CDS of *BoaMYB51.2* is 990 bp encoding a protein of 329 aa (Table 1). Compared with reference sequences from BRAD, the coding sequence of *BoaMYB51.1* isolated from Chinese kale has three different nucleotides and three different amino acids (Supplementary Figure 1), while *BoaMYB51.2* has eight different nucleotides and six different amino acids (Supplementary Figure 2). Comparison of the coding sequence with their corresponding genomic sequences revealed that both *BoaMYB51* genes consisted of three exons and two introns (Table 1).

**Table 1:** Summary of the BoaMYB51 gene sequences.

**Gene ID**	**Coding sequence (bp)**	**Protein (aa)**	**No. of exons (size in bp)**	**No. of introns (size in bp)**
*BoaMYB51.1* (Bol013207)	1002	333	3 (136,130,736)	2 (101,429)
*BoaMYB51.2* (Bol030761)	990	329	3 (136,130,724)	2 (114,234)

**Figure 1:**
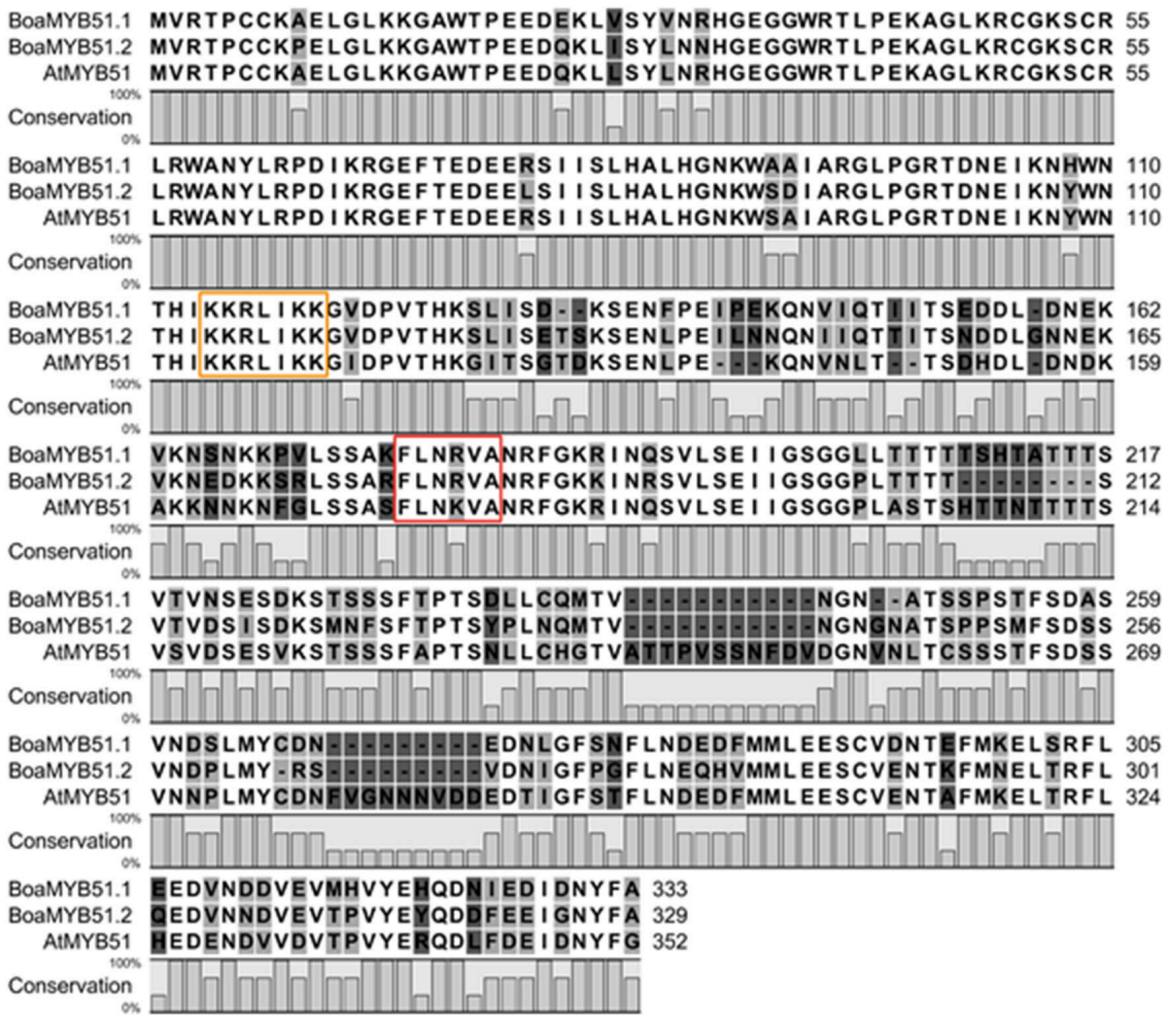
Multiple alignments of the BoaMYB51 and AtMYB51 protein sequences. Multiple alignments were performed using CLC Main Workbench software (QIAGEN). Nuclear localized signal (NLS) and characteristic “[L/F]LN[K/R]VA” motif were marked with yellow and red rectangle, respectively.

The amino acid sequences of two BoaMYB51 proteins are 76% identical and share 74 and 70% similarity with AtMYB51, respectively (Supplementary Table 3). We also found that BoaMYB51.1 protein sequence shares maximum identity (97%) with BrMYB51.2 (Bra016553), and BoaMYB51.2 protein sequence showed the highest level of identity (94%) with BrMYB51.3 (Bra025666) (Supplementary Table 3). Furthermore, amino acid sequence alignment of two BoaMYB51s with AtMYB51 demonstrated a conserved N-terminal region (Figure 1), containing R2R3-MYB domain which was predicated to serve as DNA binding domain (Dubos et al., 2010). Nuclear localized signal (NLS) and characteristic “[L/F]LN[K/R]VA” motif belonging to R2R3-type MYB subgroup 12 were also found in BoaMYB51s (Figure 1). Compared with the highly conserved N-terminal domain, the C-terminal region of BoaMYB51 exhibited conservation in patches (Figure 1).

**Figure 2:**
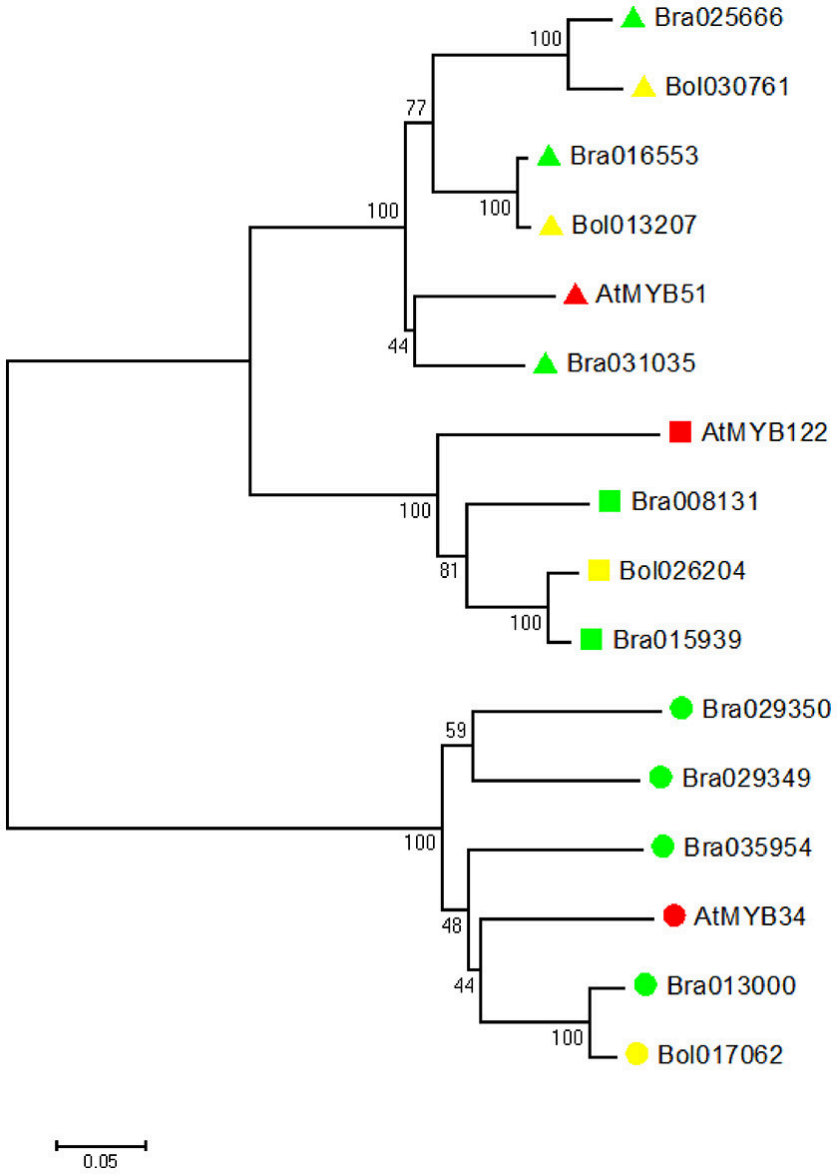
Evolutionary relationships of MYB34, MYB51, and MYB122 proteins in *A. thaliana, B. rapa* and *B. oleracea*. Phylogenetic analysis was performed using the neighbor-joining method (bootstrap test with 1000 replicates). Triangle indicates MYB51, square indicates MYB122, and circle indicates MYB34. *A. thaliana* proteins are labeled with red, *B. rapa* proteins are labeled with green, and *B. oleracea* proteins are labeled with yellow.

To investigate the evolutionary relationship, we identified all indole GSL-related *MYB* genes (*MYB34, MYB51*, and *MYB122*) in *B. rapa, B. oleracea* and *A. thaliana*. We constructed a phylogenetic tree of all deduced MYB amino acid sequences using the neighbor-joining method (Figure 2). Phylogenetic analyses revealed that in *B. rapa* genome there are three *MYB51* orthologs, four *MYB34* orthologs, and two *MYB122* orthologs, while in *B. oleracea* genome, there are two *MYB51* orthologs, one *MYB34* ortholog, and one *MYB122* ortholog (Figure 2).

### *BoaMYB51* genes exhibit overlapping but distinct expression profiles in Chinese kale

**Figure 3:**
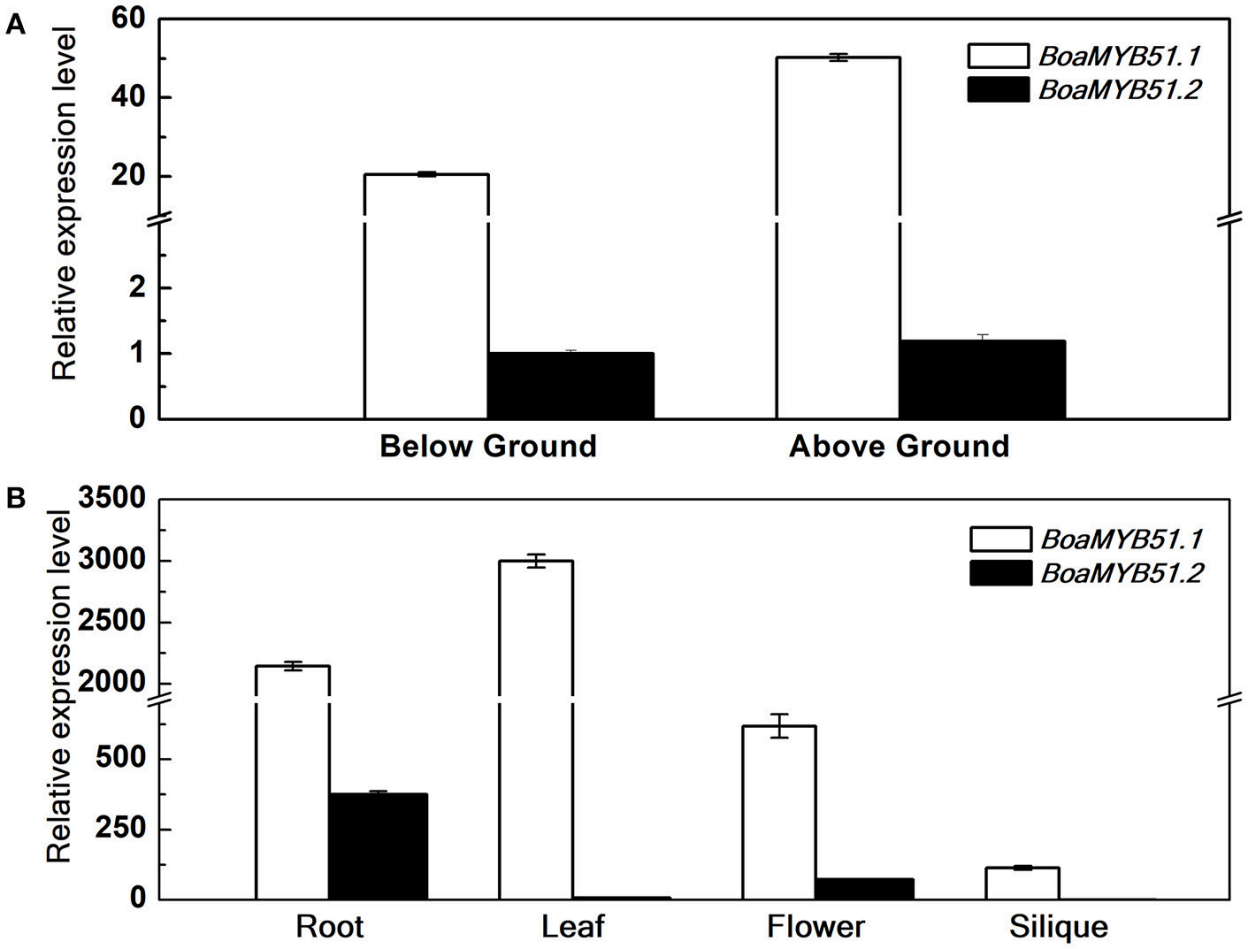
Relative gene expression of *BoaMYB51s* in Chinese kale organs at the seedling stage (5 day) **(A)** and reproductive stage (6 month) **(B)**. Each data point represents the mean of three independent biological replicates (mean ± SE). **(A)** Values are shown compared with expression level of *BoaMYB51.2* in below ground tissues. **(B)** Values are shown compared with expression level of *BoaMYB51.2* in siliques.

Distinct expression profiles among homologous genes in *Brassica* crops have been observed in many studies (Augustine et al., 2013; Zhang et al., 2015; Nour-Eldin et al., 2017). To test if two *BoaMYB51* genes are differentially expressed, qRT-PCR analysis was performed to measure the expression levels of the two genes during different developmental stages and among different tissues in Chinese kale. In general, two *BoaMYB51* genes showed overlapping expression profiles in Chinese kale and *BoaMYB51.1* was more highly expressed (Figures 3A,B). At seedling stage, *BoaMYB51.1* as well as *BoaMYB51.2* was expressed in both shoots and roots, and *BoaMYB51.1* exhibited a higher expression level than *BoaMYB51.2* (Figure 3A). At reproductive stage, *BoaMYB51* gene expression levels differed among different tissues. *BoaMYB51.1* was highly expressed in roots, leaves and flowers with the highest level in leaves while lowest level in siliques. However, *BoaMYB51.2* was expressed abundantly only in roots and flowers, and a trace accumulation of *BoaMYB51.2* transcript was detected in leaves and siliques (Figure 3B). In order to confirm the result of qRT-PCR analysis, we performed the GUS histochemical analysis of *BoaMYB51*_*pro*_*:GUS* transgenic lines in *Arabidopsis*. As shown in Supplementary Figure 3, both two *BoaMYB51* genes were expressed in aerial part and underground part, and a higher GUS staining was observed in *BoaMYB51.1*_*pro*_*:GUS*.

### *BoaMYB51s* respond to signaling molecules

**Figure 4:**
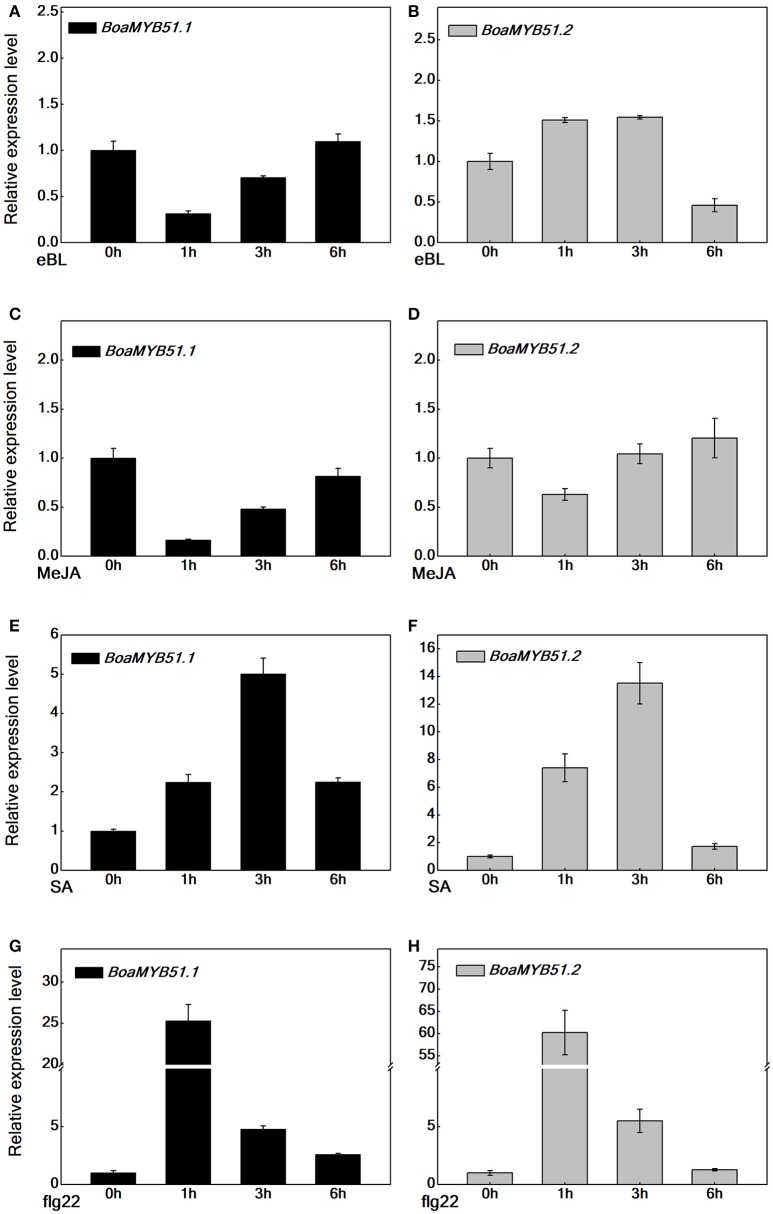
Time-course expression of *BoaMYB51s* in response to eBL, MeJA, SA, and flg22 treatments. Five-day-old Chinese kale sprouts were treated with 1 μM eBL **(A,B)**, 100 μM MeJA **(C,D)**, 100 μM SA **(E,F)**, and 100 nM flg22 **(G,H)** for indicated times before total RNAs were extracted for real time-qPCR assays. Values are shown compared with gene expression level at 0 h. Each data point represents the mean of three independent biological replicates (mean ± SE).

The transcript level of *MYB51* varies in response to phytohormones (Frerigmann and Gigolashvili, 2014). To address whether two *BoaMYB51s* respond to signaling molecules such as eBL, MeJA, SA, and flg22, we examined the expression patterns of both two genes in Chinese kale sprouts, which had been treated with corresponding signaling molecules. Generally, eBL and MeJA treatment caused a repression of *BoaMYB51s*, whereas SA and flg22 treatment induced the expression of *BoaMYB51* genes (Figures 4A–H). eBL and MeJA repressed the expression of *BoaMYB51.1* in a similar mode, which differed from the expression pattern of *BoaMYB51.2*. The expression level of *BoaMYB51.2* was reduced at 6 h under eBL treatment while at 1h with MeJA treatment (Figures 4A–D). In contrast, SA and flg22 treatment caused a significant induction of both *BoaMYB51.1* and *BoaMYB51.2* in a similar pattern. BoaMYB51s reached the highest level at 3 h with SA treatment while the peak time was 1 h after treatment with flg22 (Figures 4E–H).

### Carboxy-end is required for transcriptional activation activity of BoaMYB51s

**Figure 5:**
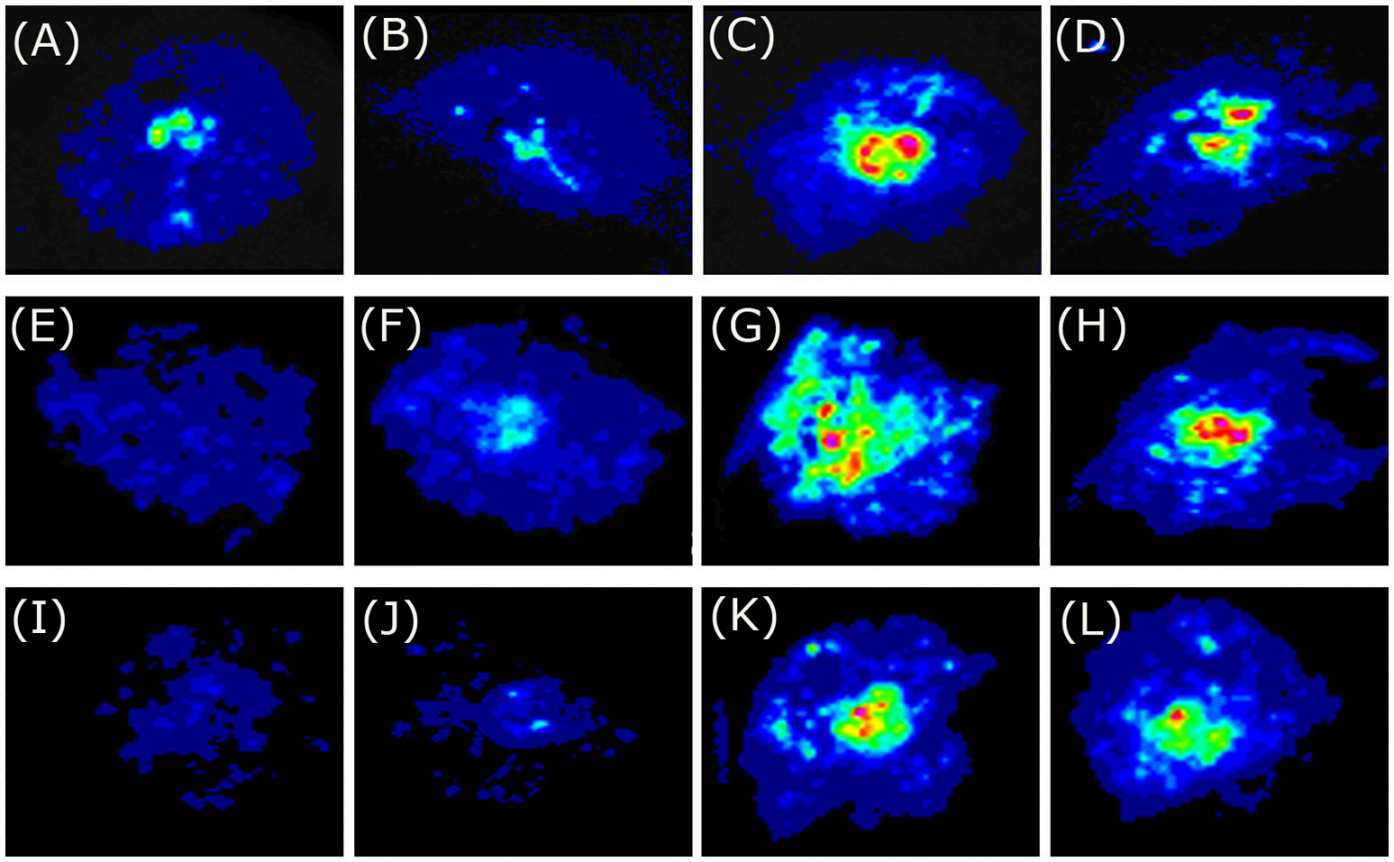
Tobacco transient expression assays showing that BoaMYB51 trans-activates the expressions of Chinese kale indole glucosinolate biosynthetic genes. **(A–D)** Co-infiltration of *BoaCYP79B2.1*_*pro*_*:LUC* and *35S*_*pro*_*:YFP, 35S*_*pro*_*:BoaMYB28.1, 35S*_*pro*_*:BoaMYB51.1* or *35S*_*pro*_*:BoaMYB51.2*, respectively. **(E–H)** Co-infiltration of *BoaCYP83B1*_*pro*_*:LUC* and *35S*_*pro*_*:YFP, 35S*_*pro*_*:BoaMYB28.1, 35S*_*pro*_*:BoaMYB51.1* or *35S*_*pro*_*:BoaMYB51.2*, respectively. **(I–L)** Co-infiltration of *BoaSOT16.1*_*pro*_*:LUC* and *35S*_*pro*_*:YFP, 35S*_*pro*_*:BoaMYB28.1, 35S*_*pro*_*:BoaMYB51.1* or *35S*_*pro*_*:BoaMYB51.2*, respectively. Representative images of *N. benthamiana* leaves 72 h after infiltration are shown.

**Figure 6:**
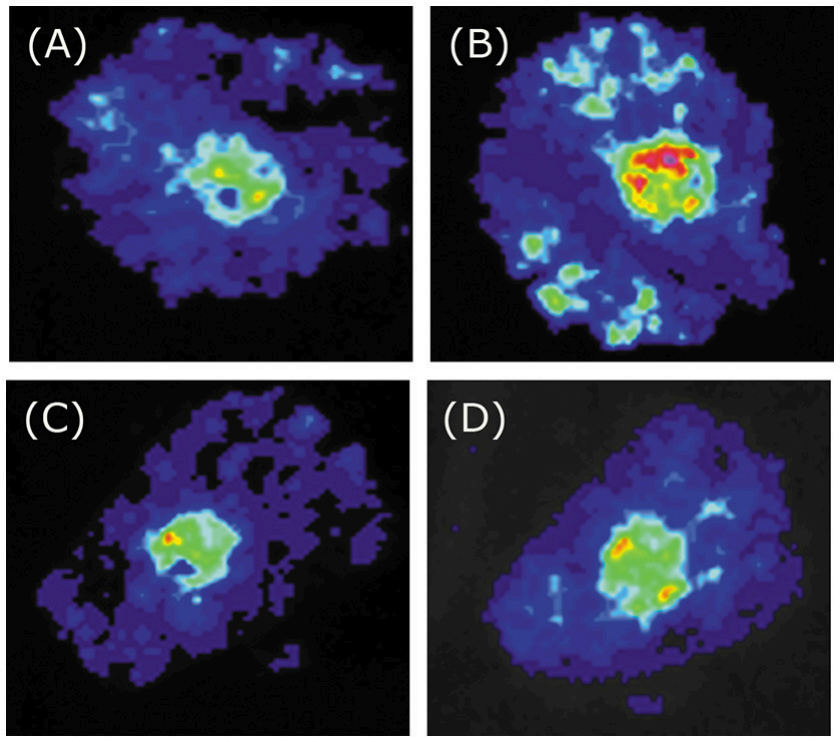
Tobacco transient expression assays showing that BoaMYB51 fail to trans-activates the expressions of *BoaCYP79F1*. **(A–D)** Co-infiltration of *BoaCYP79F1*_*pro*_*:LUC* and *35S*_*pro*_*:YFP, 35S*_*pro*_*:BoaMYB28.1, 35S*_*pro*_*:BoaMYB51.1* or *35S*_*pro*_*:BoaMYB51.2*, respectively. Representative images of *N. benthamiana* leaves 72 h after infiltration are shown.

To investigate the biological function of BoaMYB51s in Chinese kale, we analyzed the transcriptional activation ability of the two transcription factors using transient assays. *N. benthamiana* leaves were infiltrated with Agrobacteria strain GV3101 carrying the *35S*_*pro*_*:BoaMYB51* or *35S*_*pro*_*:BoaMYB28.1* construct as effector expression and reporter constructs containing the promoter of indole GSL biosynthetic genes (*BoaCYP79B2.1, BoaCYP83B1*, and *BoaSOT16.1*) or aliphatic GSL biosynthetic gene (*BoaCYP79F1*) fused with *LUC*. We showed that all tested promoters of indole GSL biosynthetic genes were activated by two BoaMYB51s but not affected by BoaMYB28.1 (Figures 5A–L and Supplementary Figures 4A–D). On the contrary, co-transformation of *35S*_*pro*_*:BoaMYB51* with *BoaCYP79F1*_*pro*_*:LUC* failed to cause an obvious increase of luminescence intensity (Figures 6A–D and Supplementary Figures 5A,B). These results implicated that BoaMYB51s are specifically involved in the transcriptional regulation of indole GSL biosynthesis in Chinese kale.

**Figure 7:**
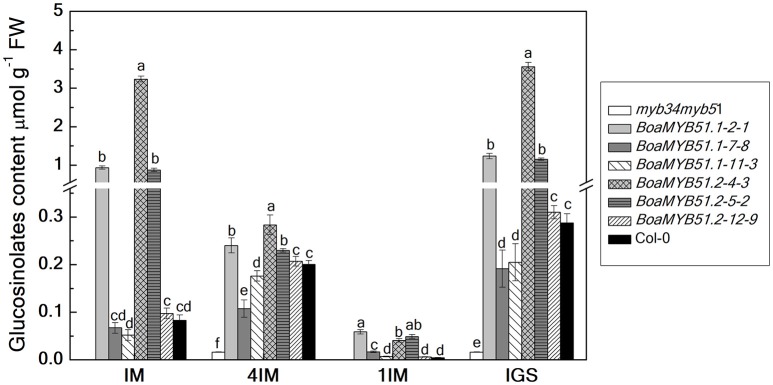
Functional complementation analyses of *BoaMYB51* genes in *Arabidopsis thaliana myb34myb51*. The glucosinolate content and profile were determined in 6-week-old rosette leaves. Three independent mutant-complemented lines for each *BoaMYB51* gene were analyzed. Each data point represents the mean of three independent biological replicates (mean ± SE).

To substantiate the functional contribution of each BoaMYB51 to the regulation on indole GSL biosynthesis, two *BoaMYB51s* were over-expressed in the *Arabidopsis* mutant *myb34myb51*. Three independent homozygous lines of each *BoaMYB51* were analyzed for total as well as individual indole GSL contents in 6-week old rosette leaves. We showed that both two *BoaMYB51* genes complemented not only the accumulation of total indole GSL contents but also individual indole GSL fractions (Figure 7). The increased indole GSL accumulation in transgenic lines correlated with increased mRNA levels of the indole GSL pathway genes of *Arabidopsis*. As shown in Supplementary Figure 6, the gene expression levels of *CYP79B2, CYP79B3, CYP83B1*, and *SOT16* were considerably increased in complementation lines compared with mutants. Moreover, transient assay revealed that both two BoaMYB51 transcription factors were able to activate the expression of *AtCYP83B1*_*pro*_*:LUC* (Supplementary Figures 7A–D). Collectively, these results clearly suggested that both two *BoaMYB51* genes are active and positively regulate the indole GSL biosynthesis.

**Figure 8:**
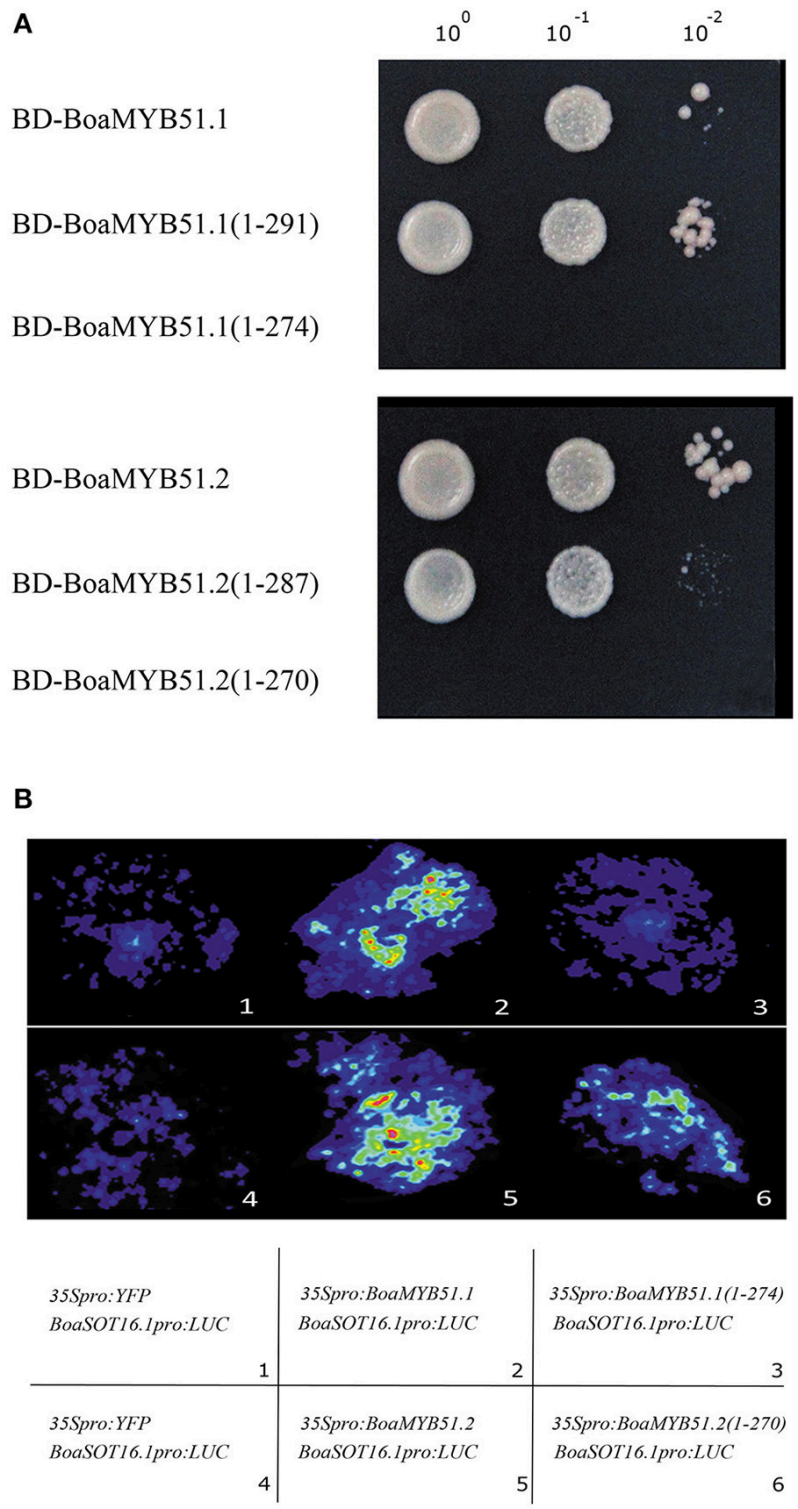
C-end is the TAD of BoaMYB51. **(A)** Yeast assay shows the deletion of C-end abolishes the transcriptional activity of BoaMYB51s. Yeast clones were grown on yeast synthetic dropout lacking Trp or on selective media lacking Ade, His, and Trp. one-tenth and one-hundred dilution yeast growth in media were also shown. **(B)** Tobacco transient expression assays show C-end is required for the transcriptional activity of BoaMYB51s. Representative images of *N. benthamiana* leaves 72 h after infiltration are shown. Each experiment was repeated at least three times with similar results.

Next, to better understand the transcriptional activation mechanism of BoaMYB51, we defined the transcriptional activation domain (TAD) of two BoaMYB51s using the MATCHMAKER GAL4-based Two-Hybrid System 3 (Clontech). In these assays, we found that both two BoaMYB51s had strong transcriptional activation activity whereas BoaMYB51.1 derivative containing amino acids from 1 to 274, and BoaMYB51.2 derivative containing amino acids from 1 to 270, do not, which suggested that the carboxy-end domain of both BoaMYB51.1 and BoaMYB51.2 could be the TAD (Figure 8A). To validate this *in planta*, we co-expressed construct encoding full-length BoaMYB51s or TAD-deleted BoaMYB51 derivatives with reporter *BoSOT16.1*_*pro*_*:LUC* in *N. benthamiana* leaves. Our results showed that BoaMYB51sΔTAD led to a substantial decrease of luminescence intensity when compared with full-length BoaMYB51s, suggesting that deletion of C-end strongly weakened the transcriptional activation activity of BoaMYB51s (Figure 8B and Supplementary Figures 8A–D). Taken together, these data indicated that C-end is required for transcriptional activation activity of BoaMYB51s.

### Interactome of BoaMYB51s identified by yeast two-hybrid screening

To identify proteins that function in cooperation with BoaMYB51, a yeast two-hybrid assay with a Chinese kale complementary DNA (cDNA) library as prey was performed. To circumvent the problem that full-length BoaMYB51s have autoactivation activity, we used BoaMYB51ΔTAD as bait. In total, we analyzed plasmids from 52 (BoaMYB51.1ΔTAD as bait) and 82 (BoaMYB51.2ΔTAD as bait) yeast colonies by sequencing. Using the basic local alignment search tool (BLAST) of BRAD, we identified a total of 34 unique inserts, most of which represent full length or nearly full-length coding sequences. Duplicates and sequences unambiguously belonging to proteins of the photosynthetic and the ribosomal machinery were excluded from further analysis, leaving a curated list of 30 candidate interactors (Table 2).

**Table 2:** List of proteins interacting with BoaMYB51 in the yeast two-hybrid screen.

**Code**	**Clones**	**Name**	**Description**
Bol036079	1	AKINBETA1, KINβ1	Encodes AKINbeta1, a subunit of the SnRK1 kinase (Sucrose non-fermenting-1-related protein kinase). Involved in regulation of nitrogen and sugar metabolism.
Bol026500	1		3-5 exonuclease domain-containing protein/K homology domain-containing protein/KH domain-containing protein
Bol025605	1	ARGININE AMIDOHYDROLASE 1	Encodes an arginase, likely to be involved in polyamine biosynthesis in pollen
Bol034826	1		P-loop containing nucleoside triphosphate hydrolases superfamily protein
Bol031300	1	BFN1, BIFUNCTIONAL NUCLEASE I, ENDO1, ENDONUCLEASE 1	Encodes a bifunctional nuclease that acts on both RNA and DNA involved in nucleic acid degradation to facilitate nucleotide and phosphate recovery during senescence. It has mismatch-specific endonuclease activity with wide recognition of single base mismatches as well as the ability to cleave indel types of mismatches
Bol014189	1	MYC2	Encodes a MYC-related transcriptional activator with a typical DNA binding domain of a basic helix-loop-helix leucine zipper motif. Binds to an extended G-Box promoter motif and interacts with Jasmonate ZIM-domain proteins.
Bol034555	1		Translation elongation factor EF1B/ribosomal protein S6 family protein
Bol040078	1	PATL3	Sec14p-like phosphatidylinositol transfer family protein
Bol025181	1		Transducin/WD40 repeat-like superfamily protein
Bol035576	2	PME3	Encodes a pectin methylesterase, targeted by a cellulose binding protein (CBP) from the parasitic nematode Heterodera schachtii during parasitism.
Bol037760	1	PLANT U-BOX 23, PUB23	Encodes a cytoplasmically localized U-box domain containing E3 ubiquitin ligase that is involved in the response to water stress and acts as a negative regulator of PAMP-triggered immunity.
Bol030761	2	MYB51	Encodes a member of the R2R3-MYB transcription family. Involved in indole glucosinolate biosynthesis.
Bol025647	1	RABG3A, RAB GTPASE HOMOLOG G3A, RABG3A	RAB GTPase homolog G3A
Bol044700	1	ALTERED SEED GERMINATION 1, ASG1	ATP-dependent DNA helicase
Bol019812	1	APG5, ATG5, AUTOPHAGY 5	Autophagy protein ATG5. Forms a conjugate with ATG12 with an essential role in plant nutrient recycling. Mutants missing ATG5 display early senescence and are hypersensitive to nitrogen or carbon starvation, accompanied by a more rapid loss of organellar and cytoplasmic proteins.
Bol029372	1	TPPA, TREHALOSE-6-PHOSPHATE PHOSPHATASE A	Homologs to the C-terminal part of microbial trehalose-6-phosphate phosphatases
Bol024700	1	HOMOLOG OF HUMAN UAP56 B, UAP56B	One of two genes encoding an ATP-dependent RNA helicase that localizes predominantly to euchromatic regions of nuclei, and associates with genes transcribed by RNA polymerase II independently from the presence of introns. It is not detected at non-transcribed loci. It interacts with ssRNA, dsRNA and dsDNA, but not with ssDNA. Its ATPase activity is stimulated by RNA and dsDNA and its ATP-dependent RNA helicase activity unwinds dsRNA but not dsDNA.
Bol026069	1	SERAT1;1, SAT-52, SAT5, SERAT1;1, SERINE ACETYLTRANSFERASE 1;1	Encodes a cytosolic serine O-acetyltransferase involved in sulfur assimilation and cysteine biosynthesis. Expressed in the vascular system
Bol030585	1	POLYUBIQUITIN 10, UBI10, UBIQUITIN 10, UBQ10	These genes encode the highly conserved 76-amino acid protein ubiquitin that is covalently attached to substrate proteins targeting most for degradation. Polyubiquitin genes are characterized by the presence of tandem repeats of the 228 bp that encode a ubiquitin monomer. Induced by salicylic acid. Independent of NPR1 for their induction by salicylic acid. The mRNA is cell-to-cell mobile.
Bol032979	1		Zinc ion binding protein
Bol026467	1	AGO4, ARGONAUTE 4, OCP11,	AGO4 is a member of a class of PAZ/PIWI domain containing proteins involved in siRNA mediated gene silencing.Loss of function mutations have reduced site specific CpNpG and CpHpH methylation and increased susceptibility to bacterial pathogens including Tobacco rattle virus
Bol008799	2		Pyruvate kinase family protein
Bol038054	2	PUX7, PLANT UBX DOMAIN-CONTAINING PROTEIN 7, PUX7	Encodes a nuclear UBX-containing protein
Bol022070	1		Unkonwn
Bol044051	2		RING/U-box superfamily protein
Bol036269	1	REPLICATION PROTEIN A 1D, RPA1D, RPA70D	Replication factor-A protein 1-like protein
Bol028834	3	FK506-BINDING PROTEIN 16-2, FKBP16-2	FK506-BINDING PROTEIN 16-2
Bol005163	2		Calcium-binding EF-hand family protein
Bol013491	1	ZW10	Homologs to Drosophila ZW10, a centromere/kinetochore protein involved in chromosome segregation
Bol043819	2	BIM1	Encodes a basic helix-loop-helix (bHLH) family protein BIM1 (BES1-INTERACTING MYC-LIKE 1)

Among the proteins interacting with MYB51 were transcription factors (Bol014189, Bol030761, and Bol043819), other nuclear-localized regulatory proteins (Bol044700, and Bol024700, and Bol026467), protein regulatory proteins (Bol037760, Bol030585, and Bol044051), and a protein with unassigned functions (Bol022070). In the current study, we further analyzed the interaction between BoaMYB51 and BoaBIM1.1 (Bol043819), because previous studies demonstrated that MYB-bHLH protein complex is important for transcriptional regulation of GSL biosynthesis (Schweizer et al., 2013; Frerigmann et al., 2014).

### BoaMYB51s interact with BoaBIMs

**Figure 9:**
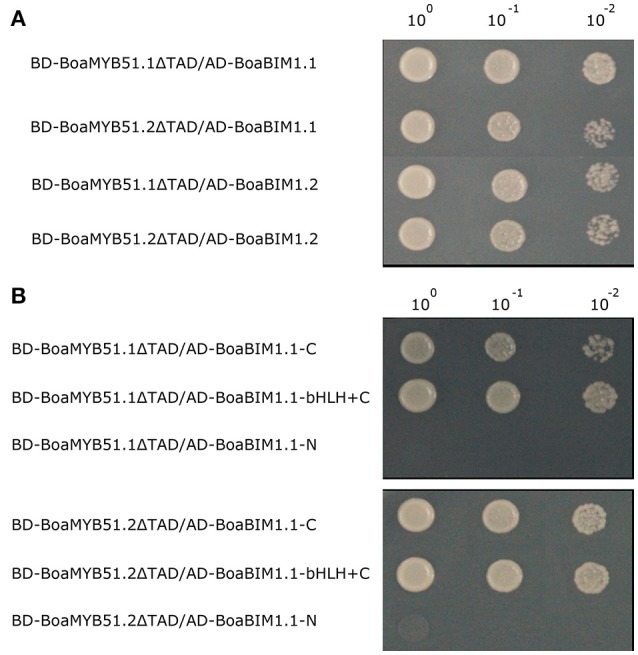
BoaMYB51s interact with BoaBIMs. **(A)** A yeast two-hybrid assay shows the interaction between BoaMYB51s and BoaBIMs. **(B)** C-end of BoaBIM1.1 was sufficient for its interaction with BoaMYB51s. BoaBIM1.1-C, C-terminal domain of BoaBIM1.1 (332–545), BoaBIM1.1-bHLH+C, bHLH and C-terminal domain of BoaBIM1.1 (278–545), and BoaBIM1.1-N, N-terminal domain of BoaBIM1.1 (1–277). Yeast clones were grown on yeast synthetic dropout lacking Trp and Leu or on selective media lacking Ade, His, Trp, and Leu. One-tenth and one-hundred dilution yeast growth in media were also shown. Each experiment was repeated at least three times with similar results.

BES1-interacting Myc-like1 (BIM1), a basic helix-loop-helix (bHLH) transcription factor, was first identified by yeast two-hybrid assay using BES1 as bait in *Arabidopsis*. BIM1 was reported to be involved in brassinosteroid responses such as hypocotyl elongation (Yin et al., 2005). There are two BIM1 paralogs in Chinese kale, namely *BoaBIM1.1* (Bol043819) and *BoaBIM1.2* (Bol008832), and *BoaBIM1.2* showed a higher expression level than *BoaBIM1.1* (Supplementary Figure 9). Screened by yeast two-hybrid assay, BoaBIM1.1 was identified to be a protein interactor of BoaMYB51s. To verify this new identified MYB-bHLH complex, we cloned full-length *BoaBIM1.1* and *BoaBIM1.2* from Chinese kale and fused them to the GAL4-activation domain. We found that both BoaBIM1.1 and BoaBIM1.2 showed interaction with two BoaMYB51s (Figure 9A). Next, we mapped the interaction domain of BoaBIM1.1 with BoaMYB51s using yeast two-hybrid assays. We found that C-end but not N-end of BoaBIM1.1 was required for its interaction with BoaMYB51s (Figure 9B).

## Discussion

Chinese kale is abundant in GSL accumulation and MYB51 acts as an important regulator of indole GSL biosynthesis. Here, we isolated both two *MYB51* homologs for the first time in *Brassica* crops. BoaMYB51s exhibited overlapping but distinct expression profiles in Chinese kale and *BoaMYB51.1* exhibited a higher expression level than *BoaMYB51.2* (Figure 3). Previous study in *Arabidopsis* indicated that MYB51 seems to be the major regulator of indole GLS biosynthesis in shoots, while MYB34 is decisive for the production of indole GLS in roots (Frerigmann and Gigolashvili, 2014). In our current research, *BoaMYB51.1* has a higher transcript level in shoots when compared to roots (Figure 3A), which was in line with the study in *Arabidopsis*. Next, we found that two *BoaMYB51* genes responded to signaling molecules in a similar pattern, which were repressed by MeJA while induced by SA and flg22 (Figure 4). In *Arabidopsis*, the expression level of *MYB51* has also been demonstrated to be activated by SA and flg22 (Millet et al., 2010; Frerigmann and Gigolashvili, 2014), while was decreased upon MeJA and eBL treatment (Guo et al., 2013; Frerigmann and Gigolashvili, 2014). However, *MYB34* gene expression was repressed by SA but triggered by MeJA (Frerigmann and Gigolashvili, 2014). To analyze whether these signaling molecules affect indole GSL accumulation, we measure the GSL content of Chinese kale sprouts after signaling molecules treatment. Our results revealed that flg22 and SA treatment elevated the content of indole GSL, while eBL treatment reduced the accumulation of indole GSL, thus demonstrating the importance of BoaMYB51.1 for signal-mediated indole GSL regulation (Supplementary Figure 10). However, MeJA treatment also increased the content of indole GSL, substantiating the importance of MYB34 upon JA signaling (Supplementary Figure 10). Collectively, it seems that BoaMYB51.1 act as a major regulator of indole GSL biosynthesis while BoaMYB51.2 perform as an accessory role. To obtain insights into BoaMYB51s, we analyzed their transcriptional activation mechanisms, defined their transcriptional activation domains, and identified BoaMYB51-interacting proteins.

### MYB51-mediated regulation on indole GSL biosynthesis is conserved

In the present study, complementation experiments in *A. thaliana myb34myb51* double mutants and tobacco transient expression assay demonstrated that both two BoaMYB51 proteins are involved in controlling indole GSL biosynthesis in Chinese kale. Two BoaMYB51 proteins were shown to exclusively trans-activate the indole GSL biosynthetic genes including *BoaCYP79B2, BoaCYP83B1* and *BoaSOT16* (Figure 5) while they failed to induce the aliphatic GSL biosynthetic gene *BoaCYP79F1* (Figure 6), which is in consistency with previous report of AtMYB51 (Gigolashvili et al., 2007b). Moreover, it has been revealed that two BoaMYB51s also activates *AtCYP83B1* (Supplementary Figures 7A–D), implying that MYB51-mediated transcription regulation of indole GSL biosynthesis is conserved. Amino acid sequence alignment of two BoaMYB51 and AtMYB51 proteins showed conserved N-terminal domain among them (Figure 1). It is known that N-terminal domain of R2R3-MYB is responsible for its DNA binding function (Dubos et al., 2010), which explains the conservation of MYB51-mediated regulation on GSL biosynthesis. Besides, when promoter sequences of *BoaSOT16.1* and *AtSOT16* genes were aligned, a conserved *cis-*element was observed (Supplementary Figure 11), which is a putative binding site of MYB51. In addition to indole GSL regulator, we also found that BoaMYB28 positively regulates aliphatic GSL biosynthetic gene without directly affecting indole GSL biosynthesis in Chinese kale, which is in line with the results in *A. thaliana* and other *Brassica* crops (Gigolashvili et al., 2007a; Augustine et al., 2013), suggesting that the MYB-mediated regulatory mechanism of aliphatic GSL and indole GSL biosynthesis is conserved in *Brassicaceae*. To further study the MYB-mediated specific regulation on GSL biosynthesis, we swapped the N-terminal fragments of BoaMYB28 for the N-terminal domain of BoaMYB51.1 and co-expressed this chimeric protein with *BoaSOT16.1*_*pro*_*:LUC* and *BoaCYP79F1*_*pro*_*:LUC*, respectively in tobacco leaves (Supplementary Figure 12). Our results revealed that BoaMYB51.1^N−SWAP^ protein failed to activate the promoter of *BoaSOT16.1*, whereas co-transfomation with *BoaCYP79F1*_*pro*_*:LUC* led to a significant increase of luminescence intensity (Supplementary Figure 12). We also generated another chimeric protein with N-terminal fragments of BoaMYB51.1 and C-terminal fragments of BoaMYB28 and this BoaMYB51.1^C−SWAP^ protein showed an opposite pattern in tobacco transient assay when compared with BoaMYB51.1^N−SWAP^ (Supplementary Figure 12). Collectively, these results suggest that the N-terminal domain is critical for the specific regulation of GSL biosynthesis.

### C-end is the transcriptional activation domain (TAD) of BoaMYB51s

Typically, a TF consists of a DNA-binding domain (DBD) and a transcription regulatory domain. It is generally considered that plant R2R3-MYB TFs share a conserved N-terminal MYB DBD, and C-terminal part of the protein usually harbors an activation or repression domain (Dubos et al., 2010). However, the experimentally proven details about TAD in R2R3-MYB TFs is limited. From studies by Goff et al. (1992), the TAD of the ZmC1 is located in a carboxy-terminal acidic region. Moreover, the C-terminal acidic region of AtMYB2 and AtMYB12 was found to be able to activate transcription (Urao et al., 1996; Stracke et al., 2017). Here, we identified the TAD of BoaMYB51, which is also located in the C-terminal region, by using yeast two-hybrid system and tobacco transient assay (Figures 8A,B). According to previous reports, the TAD of a TF often contains acidic, glutamine-rich, or proline-rich stretches of amino acids (Stracke et al., 2017). The TAD of BoaMYB51 defined in this study is enriched in acidic amino acids, but proline-rich or glutamine-rich amino acid stretches were not identified, which is in good accordance with the features of the TADs of above-mentioned R2R3-MYB TFs. It is a general phenomenon that the acidic TADs of TFs overlap closely with the destruction elements (Salghetti et al., 2000). Whether the acidic TAD of BoaMYB51 also functions as a degron which may account for the unstable characteristic of MYB51 protein needs further analysis. Since the amino sequences between AtMYB51 and BoaMYB51 are highly conserved, we also identified the TAD of AtMYB51 based on sequence alignment which is located in the amino acid region 294 to 352, and the result was validated by yeast two-hybrid system (Supplementary Figure 13). Furthermore, we also found that the TAD of AtMYB28 and BoaMYB28.1 are localized to C-end (data not shown), indicating that the C-terminally localized TAD is shared as a conserved feature by GSL-related MYBs.

### MYB51-BIM1 protein complex might be involved in regulating indole GSL biosynthesis

The bHLH protein BIM1 is a brassinosteroid signaling component involved in mediating BR-responsive gene expression. It has been demonstrated that BIM1 regulates hypocotyl elongation, embryo patterning, and UVR8-mediated photomorphogenesis via interacting with the atypical bHLH protein BES1, AP2 protein DRN and UV-B light photoreceptor UVR8, respectively (Yin et al., 2005; Chandler et al., 2009; Liang et al., 2018). Here, BoaBIM1.1 was identified from a two-hybrid screen using BoaMYB51.1ΔTAD as bait (Table 2), and protein-protein interaction between BoaBIM1s and BoaMYB51s has been further confirmed. Gene expression analysis revealed that both *BoaBIM1.1* and *BoaBIM1.2* are expressed in Chinese kale leaves at a relatively high level (Supplementary Figure 9), in which BoaMYB51s are also highly expressed (Figure 3). To study the role of BIM1 on GSL biosynthesis, *Arabidopsis* triple mutant *bim123* was used for GSL and qRT-PCR analysis. We found that indole GSL content and expression levels of indole GSL biosynthetic genes *AtCYP79B2, AtCYP79B3*, and *AtCYP83B1* were decreased (Supplementary Figures 14A,B), implicating that BIM1 is a putative regulator of indole GSL biosynthesis. Besides, it has also been showed that the transcript level of *AtMYB51* was not affected in *bim123*, suggesting that BIM1 interact with MYB51 and regulate the transcription of indole GSL biosynthetic genes.

Protein-protein interaction between MYB and bHLH transcription factors is a well-known paradigm of combinatorial gene regulation in plants and are instrumental in various biological processes (Feller et al., 2011). MYB-bHLH protein complex has been revealed to regulate anthocyanin biosynthesis (Gonzalez et al., 2008) and trichome morphogenesis (Zhao et al., 2008). Besides, the bHLH protein MYC2, known as a component of JA signaling pathway, and its close homologs MYC3 and MYC4 were shown to interact with GSL-related MYBs and affect thereby the GSL biosynthesis (Schweizer et al., 2013; Frerigmann et al., 2014). In this study, we identified another MYB-bHLH protein complex involved in the GSL biosynthesis regulation. It is known that the three MYCs bind to GSL-related MYBs via an N-terminal domain, the so-called JID domain (JAZ interaction domain). However, the C-terminal domain of BoaBIM1 show interaction with BoaMYB51 in yeast two-hybrid assays (Figure 9B), suggesting that the interaction mechanism between these two MYB-bHLH protein complexes is different.

## Author contributions

CC and QW designed the research, CC and WY conducted the experiments. WY, HM, MD, MW, JL, and WZ analyzed the data. CC and QW wrote the manuscript. All authors read and approved the final manuscript.

### Conflict of interest statement

The authors declare that the research was conducted in the absence of any commercial or financial relationships that could be construed as a potential conflict of interest.
