# Supplementary material for: Functional Characterization of BoaMYB51s as Central Regulators of Indole Glucosinolate Biosynthesis in Brassica oleracea var. alboglabra Bailey
Source: Front Plant Sci. 2018 Nov 6;9:1599. doi: 10.3389/fpls.2018.01599 (PMC6232877; doi:10.3389/fpls.2018.01599)

Supplenmentary Data

Table S1 Primers used for qRT-PCR experiment.

Table S2 Primers used for construct generation.

Table S3 Amino acid sequence identities (%) of MYB51 among *B. oleracea*, *B. rapa* and *A. thaliana*.

Figure S1 Alignment of *BoMYB51.1* coding DNA sequences (CDS) (A) and deduced protein sequences (B) obtained from Chinese kale and BRAD. The sequence alignment was performed using the CLC Main Workbench software (QIAGEN). Color shade represents different residues.

Figure S2 Alignment of *BoMYB51.2* coding DNA sequences (CDS) (A) and deduced protein sequences (B) obtained from Chinese kale and BRAD. The sequence alignment was performed using the CLC Main Workbench software (QIAGEN). Color shade represents different residues.

Figure S3 Histochemical GUS staining of 10-d-old $BoaMYB51_{pro}$*:GUS* transgenic *Arabidopsis* seedlings: (A) and (C) $BoaMYB51.1_{pro}$*:GUS*; (B) and (D) $BoaMYB51.2_{pro}$*:GUS*.

Figure S4 (A) to (C) Quantitative analysis of luminescence intensity in Fig. 5. Values are means ± SE of five independent determinations. ANOVA was performed for statistical analysis; bars with different letters are significantly different from each other ($P < 0.01$). (D) qRT-PCR analysis of *BoMYB28.1*, *BoMYB51.1*, and *BoMYB51.2* expression in the infiltrated leaf areas shown in Fig. 5. Total RNA was extracted from leaves of *N. benthamiana* coinfiltrated with the indicated constructs. Values are means±SE of five independent determinations.

Figure S5 (A) Quantitative analysis of luminescence intensity in Fig. 6. Values are means ± SE of five independent determinations. ANOVA was performed for statistical analysis; bars with different letters are significantly different from each other ($P < 0.01$). (B) qRT-PCR analysis of *BoaMYB28.1*, *BoaMYB51.1*, and *BoaMYB51.2* expression in the infiltrated leaf areas shown in Fig. 6. Total RNA was extracted from leaves of *N. benthamiana* coinfiltrated with the indicated constructs. Values are means±SE of five

independent determinations.

Figure S6 Relative gene expression of *CYP79B2, CYP79B3, CYP83B1,* and *SOT16* in 10-d-old seedlings of *BoaMYB51* transgenic complementation lines. Expression data are presented relative to *Actin2*. Each data point represents the mean of three independent biological replicates (mean±SE).

Figure S7 (A) Tobacco transient expression assays showing that BoMYB51 trans-activates the gene expression of *AtCYP83B1*. The right panel indicates the infiltrated constructs. Representative images of *N. benthamiana* leaves 72 h after infiltration are shown. (B) and (C) Quantitative analysis of luminescence intensity in (A). Values are means ± SE of five independent determinations and *$P<0.05$ by Student's *t*-test. (D) qRT-PCR analysis of *BoaMYB51.1* and *BoaMYB51.2* expression in the infiltrated leaf areas shown in (A). Total RNA was extracted from leaves of *N. benthamiana* coinfiltrated with the indicated constructs. Values are means±SE of five independent determinations.

Figure S8 (A) and (B) Quantitative analysis of luminescence intensity in Fig. 8B. Values are means ± SE of five independent determinations. ANOVA was performed for statistical analysis; bars with different letters are significantly different from each other ($P < 0.01$). (C) qRT-PCR analysis of *BoaMYB51.1* expression in the infiltrated leaf areas shown in Fig. 8B. (D) qRT-PCR analysis of *BoaMYB51.2* expression in the infiltrated leaf areas shown in Fig. 8B. (C) and (D) Total RNA was extracted from leaves of *N. benthamiana* coinfiltrated with the indicated constructs. Values are means±SE of five independent determinations.

Figure S9 Relative gene expression of *BoaBIM1s* in Chinese kale organs at reproductive stage (6 month). Each data point represents the mean of three independent biological replicates (mean±SE). Values are shown compared with expression level of *BoBIM1.1* in flowers.

Figure S10 The effect of eBL, MeJA, SA, and flg22 treatment on the glucosinolate contents in Chinese kale sprouts. Five-day-old Chinese kale sprouts were treated with 1 μM eBL, 100 μM MeJA, 100 μM SA, and 100 nM flg22 for 24h before glucosinolate analysis. Each data is the mean (± standard error) of three replicates (n = 3). ANOVA

was performed for statistical analysis, and values not sharing the same letter are significantly different at $p < 0.05$.

Figure S11 Nucleotide alignment of the *BoSOT16.1* and *AtSOT16* promoter sequences. MYB binding *cis*-elements are highlighted in light blue (ACC(A/T)A(A/C)) and green (CNGTT(A/G) or GTT(A/T)GTT(A/G)). Nucleotide alignment was performed using NCBI BLAST.

Figure S12 Tobacco transient expression assays showing that N-terminal domains of BoaMYBs were critical for their specific regulation of GSL biosynthesis. (A) Schematics of BoaMYB51.1$^{N\text{-}SWAP}$ and BoaMYB51.1$^{C\text{-}SWAP}$ construct. (B) Quantitative analysis of luminescence intensity in tobacco leaves using $BoaSOT16_{pro}$*: LUC* as reporter. (C) Quantitative analysis of luminescence intensity in tobacco leaves using $BoaCYP79F1_{pro}$*: LUC* as reporter. Error bars indicate SE from at least four replicates.

Figure S13 C-end is also the TAD of AtMYB51. Yeast assay shows the deletion of C-end abolishes the transcriptional activity of AtMYB51. Yeast clones were grown on yeast synthetic dropout lacking Trp or on selective media lacking Ade, His, and Trp. one-tenth and one-hundred dilution yeast growth in media were also shown.

Figure S14 (A) The Indole GLS content of *bim123*. The glucosinolate content and profile were determined in 2-week-old seedlings. Each data point represents the mean of three independent biological replicates (mean±SE) and *P<0.05 by Student's *t*-test. (B) Relative gene expression of *CYP79B2, CYP79B3,* and *CYP83B1* in 10-d-old seedlings of *bim123*. Expression data are presented relative to *Actin2*. Each data point represents the mean of three independent biological replicates (mean±SE).

Table S1 primers used for qRT-PCR experiments

Primer Name	Primes sequence (5'-3')
RT- BoaACTIN2-F	TGGCTGAGGCTGATGACATTC
RT- BoaACTIN2-R	TACCTCTCTTGGACTGTGCCT
RT- BoaMYB51.1-F	GTTCTTAAACAGAGTAGCTAATAGGTTC
RT- BoaMYB51.1-R	TTCGGAGTTAACGGTGACACTTG
RT- BoaMYB51.2-F	CCTTCACGGCAACAAATGGTCTGAT
RT- BoaMYB51.2-R	CGGGTCGACACCTTTCTTGATCAA
RT- BoaMYB28.1-F	GTTGCGGCTAAGGTCACTTCT
RT- BoaMYB28.1-R	CAGAAGTAGCGTTGATCTCATGC
RT-BoBIM1.1-F	ATGGAATCACGAACCTGC
RT-BoBIM1.1-R	CGGTCTATAACAACGCCT
RT-BoBIM1.2-F	TACCAAGGATGGAACCAAG
RT-BoBIM1.2-R	CAGACTGTTGCTTTGAACC

Table S2 primers used for construct generation

Primer Name	Primes sequence (5’-3’)
TOPO-BoaMYB51.1-F	caccATGGTGCGGACACCGTGTTG
TOPO-BoaMYB51.1-R	TGCAAAATAGTTATCAATATCT
TOPO-BoaMYB51.2-F	caccATGGTGCGGACACCATGTTG
TOPO-BoaMYB51.2-R	TGCAAAATAGTTACCAATCTCT
BoaMYB51.1pro-F	caccTAACGCTAGACCAAGTCTTTTAGGATT
BoaMYB51.1pro-R	TCTTGATTCTTGATGGGGTTGTTCTCAAG
BoaCYP79B2.1pro-F	caccGGTTTTTGGCAGGTGAAGTGGTG
BoaCYP79B2.1pro-R	GTTTTTGGGCTGAGGATAAAGAGAGAA
BoaCYP83B1$_{pro}$-F	caccATGGTGATCTTGTAGGTATCCGGT
BoaCYP83B1$_{pro}$-R	GGCGGCAATAATCAAGAAGAGATC
BoaSOT16.1$_{pro}$-F	caccTGGGGATGATGTTGAGTGATG
BoaSOT16.1$_{pro}$-R	TTATATCTCTCTCGGTTTTGTTTGATTTA
BoaCYP79F1$_{pro}$-F	caccGATTATAACGGGAAAACCAAAAACATCAATAGTTG
BoaCYP79F1$_{pro}$-R	GACATATTTGTGCCTAGGACGAGTCAT
AtCYP83B1$_{pro}$-F	caccGAGGAAGATTTGACAGAAAC
AtCYP83B1$_{pro}$-R	ACCGGCTATAATCAATAAGAGATCC
SmaI-BoaMYB51.1-F	acgggggactctagacccgggATGGTGCGGACACCGTGTT
BamHI-BoaMYB51.1-R	cgtgggtccactagtggatccTGCAAAATAGTTATCAATATCTTCGATA
SmaI-BoaMYB51.2-F	acgggggactctagacccgggATGGTGCGGACACCATGTTG
BamHI-BoaMYB51.2-R	cgtgggtccactagtggatccTGCAAAATAGTTACCAATCTCTTCG
SmaI-BoaMYB28.1-F	acgggggactctagacccgggATGTCAAGAAAACCGTGTTGTGTC
BamHI-BoaMYB28.1-R	cgtgggtccactagtggatccTATGAGGGTATCAGACTCCGTGTC

BamHI-BoaMYB51.1-274-R	cgtgggtccactagtggatccTCCGAGATTATCCTCATTATCACAGT
BamHI-BoaMYB51.2-270-R	cgtgggtccactagtggatccTCCGATATTATCAACACTACGGTACA
BD- BoaMYB51.1-F	atggccatggaggccgaattcATGGTGCGGACACCGTGTT
BD- BoaMYB51.1-R	ctagttatgcggccgctgcagTGCAAAATAGTTATCAATATCTTCGATA
BD- BoaMYB51.2-F	atggccatggaggccgaattcATGGTGCGGACACCATGTTG
BD- BoaMYB51.2-R	ctagttatgcggccgctgcagTGCAAAATAGTTACCAATCTCTTCG
BD- BoaMYB51.1-274-R	ctagttatgcggccgctgcagTCCGAGATTATCCTCATTATCACAGT
BD- BoaMYB51.1-291-R	ctagttatgcggccgctgcagACAAGACTCTTCCAACATCATGAAAT
BD- BoaMYB51.2-270-R	ctagttatgcggccgctgcagTCCGATATTATCAACACTACGGTACA
BD- BoaMYB51.2-287-R	ctagttatgcggccgctgcagACAAGACTCTTCCAACATCATGACA
AD-BoBIM1-F	gtaccagattacgctcatatgATGGAGCTTCCTCAACCTCGT
AD-BoBIM1.1-R	cagctcgagctcgatggatccACTTGTGTTGTTGTTGTTCGTCTTC
AD-BoBIM1.2-R	cagctcgagctcgatggatccACTTGTATTGCACGTTTTGAGCC
AD-BoBIM1.1-277-R	cagctcgagctcgatggatccTTGATCGTTACCAACACCTCTCG
AD-BoBIM1.1-278-F	gtaccagattacgctcatatgAAGCTGAACACGCCTAGGTCC
AD-BoBIM1.1-332-F	gtaccagattacgctcatatgCAGGAGAAAACAAGCAAGTACGAA

Table S3 Amino acid sequence identities (%) of MYB51 among *B. oleracea*, *B. rapa* and *A. thaliana*.

	BoMYB51.1	BoMYB51.2	BrMYB51.1	BrMYB51.2	BrMYB51.3	AtMYB51
BoMYB51.1 (Bol013207)	***	76	80	97	78	74
BoMYB51.2 (Bol030761)		***	72	74	94	70
BrMYB51.1 (Bra031035)			***	81	74	71
BrMYB51.2 (Bra016553)				***	79	75
BrMYB51.3 (Bra025666)					***	71
AtMYB51 (AT1G18570)						***

Supplementary Figure S1

(A)

```
BoaMYB51.1(Chinese kale)  ATGGTGCGGACACCGTGTTGCAAAGCTGAACTAGGCTTAAAGAAAGGAGCATGGACTCCC 60
Bol013207(BRAD)           ATGGTGCGGACACCGTGTTGCAAAGCTGAACTAGGCTTAAAGAAAGGAGCATGGACTCCC 60

BoaMYB51.1(Chinese kale)  GAGGAAGATGAGAAGCTTGTCTCCTACGTCAACCGTCACGGTGAAGGTGGATGGCGAACT 120
Bol013207(BRAD)           GAGGAAGATCAGAAGCTTGTCTCCTACGTCAACCGTCACGGTGAAGGTGGATGGCGAACT 120

BoaMYB51.1(Chinese kale)  CTCCCCGAAAAAGCTGGACTCAAAAGATGTGGCAAAAGCTGCAGACTGAGATGGGCTAAT 180
Bol013207(BRAD)           CTCCCCGAAAAAGCTGGACTCAAAAGATGTGGCAAAAGCTGCAGACTGAGATGGGCTAAT 180

BoaMYB51.1(Chinese kale)  TATCTACGACCTGACATCAAAAGAGGAGAGTTCACTGAAGATGAAGAACGTTCTATCATC 240
Bol013207(BRAD)           TATCTACGACCTGACATCAAAAGAGGAGAGTTCACTGAAGATGAAGAATGTTCTATCATC 240

BoaMYB51.1(Chinese kale)  TCTCTTCATGCCCTTCATGGCAACAAATGGGCTGCAATAGCTCGTGGATTACCGGGAAGA 300
Bol013207(BRAD)           TCTCTTCATGCCCTTCATGGCAACAAATGGGCTGCAATAGCTCGTGGATTACCGGGAAGA 300

BoaMYB51.1(Chinese kale)  ACCGATAACGAAATCAAGAACCACTGGAACACTCATATCAAAAAACGTTTGATAAAGAAA 360
Bol013207(BRAD)           ACCGATAACGAAATCAAGAACCACTGGAACACTCATATCAAAAAACGTTTGATAAAGAAA 360

BoaMYB51.1(Chinese kale)  GGTGTCGATCCGGTTACACACAAGAGCTTGATTTCCGACAAATCAGAAAACTTCCCAGAG 420
Bol013207(BRAD)           GGTGTCGATCCGGTTACACACAAGAGCTTGATTTCCGACAAATCAGAAAACTTCCCAGAG 420

BoaMYB51.1(Chinese kale)  ATTCCAGAGAAGCAAAACGTTATTCAGACAATTATAACGAGTGAAGATGATCTTGATAAT 480
Bol013207(BRAD)           ATTCCAGAGAAGCAAAACGTTATTCAGACAATTATAACGAGTGAAGATGATCTTGATAAT 480

BoaMYB51.1(Chinese kale)  GAGAAGGTGAAGAATAGCAACAAGAAGCCGGTATTATCATCGGCTAAGTTCTTAAACAGA 540
Bol013207(BRAD)           GAGAAGGTGAAGAATAGCAACAAGAAGCCGGTATTATCATCGGCTAAGTTCTTAAACAGA 540

BoaMYB51.1(Chinese kale)  GTAGCTAATAGGTTCGGAAAGAGAATCAATCAAAGCGTTCTGTCTGAGATTATCGGAAGT 600
Bol013207(BRAD)           GTAGCTAATAGGTTCGGAAAGAGAATCAATCAAAGCGTTCTGTCTGAGATTATCGGAAGT 600

BoaMYB51.1(Chinese kale)  GGTGGCCTACTTACTACTACTACTACAAGTCACACTGCTACTACTACAAGTGTCACCGTT 660
Bol013207(BRAD)           GGTGGCCCACTTACTACTACTACTACAAGTCACACTGCTACTACTACAAGTGTCACCGTT 660

BoaMYB51.1(Chinese kale)  AACTCCGAATCAGATAAGTCAACTAGCTCTTCTTTCACACCAACCTCAGATCTTCTATGC 720
Bol013207(BRAD)           AACTCCGAATCAGATAAGTCAACTAGCTCTTCTTTCACACCAACCTCAGATCTTCTATGC 720

BoaMYB51.1(Chinese kale)  CAGATGACTGTTAACGGTAACGCTACATCGTCTCCGTCCACATTCTCTGATGCATCCGTT 780
Bol013207(BRAD)           CAGATGACTGTTAACGGTAACGCTACATCGTCTCCGTCCACATTCTCTGATGCATCCGTT 780

BoaMYB51.1(Chinese kale)  AATGATAGTTTAATGTACTGTGATAATGAGGATAATCTCGGATTCTCAAATTTTCTGAAC 840
Bol013207(BRAD)           AATGATAGTTTAATGTACTGTGATAATGAGGATAATCTCGGATTCTCAAATTTTCTGAAC 840

BoaMYB51.1(Chinese kale)  GATGAAGATTTCATGATGTTGGAAGAGTCTTGTGTTGACAACACTGAGTTTATGAAAGAA 900
Bol013207(BRAD)           GATGAAGATTTCATGATGTTGGAAGAGTCTTGTGTTGACAACACTGAGTTTATGAAAGAA 900

BoaMYB51.1(Chinese kale)  CTTTCGAGGTTTCTTGAGGAGGACGTGAACGACGACGTCGAGGTGATGCATGTCTATGAG 960
Bol013207(BRAD)           CTTTCGAGGTTTCTTGAGGAGGACGTGAACGACGACGTCGAGGTGATGCATGTCTATGAG 960

BoaMYB51.1(Chinese kale)  CATCAAGACAATATCGAAGATATTGATAACTATTTTGCATGA 1002
Bol013207(BRAD)           CATCAAGACAATATCGAAGATATTGATAACTATTTTGCATGA 1002
```

(B)

```
BoaMYB51-1.1(Chinese kale)  MVRTPCCKAELGLKKGAWTPEEDEKLVSYVNRHGEGGWRTLPEKAGLKRCGKSCRLRWAN 60
Bol013207(BRAD)             MVRTPCCKAELGLKKGAWTPEEDQKLVSYVNRHGEGGWRTLPEKAGLKRCGKSCRLRWAN 60

BoaMYB51-1.1(Chinese kale)  YLRPDIKRGEFTEDEERSIISLHALHGNKWAAIARGLPGRTDNEIKNHWNTHIKKRLIKK 120
Bol013207(BRAD)             YLRPDIKRGEFTEDEECSIISLHALHGNKWAAIARGLPGRTDNEIKNHWNTHIKKRLIKK 120

BoaMYB51-1.1(Chinese kale)  GVDPVTHKSLISDKSENFPEIPEKQNVIQTIITSEDDLDNEKVKNSNKKPVLSSAKFLNR 180
Bol013207(BRAD)             GVDPVTHKSLISDKSENFPEIPEKQNVIQTIITSEDDLDNEKVKNSNKKPVLSSAKFLNR 180

BoaMYB51-1.1(Chinese kale)  VANRFGKRINQSVLSEIIGSGGLLTTTTTSHTATTTSVTVNSESDKSTSSSFTPTSDLLC 240
Bol013207(BRAD)             VANRFGKRINQSVLSEIIGSGGPLTTTTTSHTATTTSVTVNSESDKSTSSSFTPTSDLLC 240

BoaMYB51-1.1(Chinese kale)  QMTVNGNATSSPSTFSDASVNDSLMYCDNEDNLGFSNFLNDEDFMMLEESCVDNTEFMKE 300
Bol013207(BRAD)             QMTVNGNATSSPSTFSDASVNDSLMYCDNEDNLGFSNFLNDEDFMMLEESCVDNTEFMKE 300

BoaMYB51-1.1(Chinese kale)  LSRFLEEDVNDDVEVMHVYEHQDNIEDIDNYFA 333
Bol013207(BRAD)             LSRFLEEDVNDDVEVMHVYEHQDNIEDIDNYFA 333
```

Supplementary Figure S2

(A)

```
BoaMYB51.2(Chinese kale)  ATGGTGCGGACACCATGTTGCAAACCTGAATTAGGGTTAAAGAAAGGAGCTTGGACTCCC 60
Bol030761(BRAD)           ATGGTGCGGACACCATGTTGCAAACCTGAATTAGGGTTAAAGAAAGGAGCTTGGACTCCC 60

BoaMYB51.2(Chinese kale)  GAGGAAGATCAGAAGCTTATCTCCTACCTTAACAATCACGGTGAAGGTGGATGGCGAACT 120
Bol030761(BRAD)           GAGGAAGATCAGAAGCTTATCTCCTACCTTAACAATCACGGTGAAGGTGGATGGCGAACT 120

BoaMYB51.2(Chinese kale)  CTACCCGAAAAAGCTGGACTGAAGAGATGTGGCAAAAGCTGCAGACTGAGGTGGGCCAAT 180
Bol030761(BRAD)           CTACCCGAAAAAGCTGGACTGAAGAGATGTGGCAAAAGCTGCAGACTGAGGTGGGCCAAT 180

BoaMYB51.2(Chinese kale)  TATCTTAGACCTGACATCAAAAGAGGAGAGTTCACTGAAGATGAAGAACTTTCTATCATC 240
Bol030761(BRAD)           TATCTAAGACCTGACATCAAAAGAGGAGAGTTCACTGAAGATGAAGAACTTTCTATCATC 240

BoaMYB51.2(Chinese kale)  TCTCTTCACGCCCTTCACGGCAACAAATGGTCTGATATAGCTCGTGGATTACCGGGAAGA 300
Bol030761(BRAD)           TCTCTTCACGCCCTTCACGGCAACAAATGGTCTGATATAGCTCGTGGATTACCGGGAAGA 300

BoaMYB51.2(Chinese kale)  ACCGATAACGAAATCAAGAACTACTGGAACACTCATATAAAAAAACGTTTGATCAAGAAA 360
Bol030761(BRAD)           ACCGATAACGAAATCAAGAACTACTGGAACACTCATATAAAAAAACGTTTGATCAAGAAA 360

BoaMYB51.2(Chinese kale)  GGTGTCGACCCGGTTACACACAAGAGTTTGATCTCCGAAACCAGCAAATCAGAAAACCTC 420
Bol030761(BRAD)           GGTGTCGACCCGGTTACACACAAGAGTTTGATCTCCGAAACCAGCAAATCAGAAAACCTC 420

BoaMYB51.2(Chinese kale)  CCGGAGATTCTTAATAATCAAAACATTATTCAGACAACTATAACGAGTAACGATGATCTT 480
Bol030761(BRAD)           CCGGAGATTCTGAATAATCAAAACATTATTCAGACAACTATATCGAGTAACGATGATCTT 480

BoaMYB51.2(Chinese kale)  GGTAATAATGAGAAGGTCAAGAATGAAGACAAGAAGTCAAGATTATCATCAGCTAGGTTC 540
Bol030761(BRAD)           GGTAATAATGAGAAGGTCAAGAATGAAGACAAGAAGTCAAGATTATCATCAGCTAGGTTC 540

BoaMYB51.2(Chinese kale)  TTGAACAGGGTAGCAAATAGGTTTGGAAAGAAAATCAATCGGAGTGTTCTCTCGGAGATT 600
Bol030761(BRAD)           TTGAACAAGGTAGCAAATAGGGTTGGAAAGAAAATCAATCAGAGTGTTCTCTCGGAGATT 600

BoaMYB51.2(Chinese kale)  ATCGGAAGTGGTGGCCCACTCACTACTACTACAAGTGTCACCGTTGACTCTATATCAGAT 660
Bol030761(BRAD)           ATCGGAAGTGGTGGCCCACTCACTACTACTACAAGTGTCACCGTTGACTCTATATCAGAT 660

BoaMYB51.2(Chinese kale)  AAGTCAATGAATTTTTCCTTCACACCAACTTCGTATCCTCTCAACCAGATGACCGTTAAT 720
Bol030761(BRAD)           AAGTCAATGAATTTTTCCTTCACACCAACTTCGTATCCTCTCAACCAGATGACCGTTAAT 720

BoaMYB51.2(Chinese kale)  GGTAACGGTAACGCTACGTCGCCTCCATCCATGTTCTCTGATTCCTCCGTTAACGATCCT 780
Bol030761(BRAD)           GATAACGGTAACGCTACGTCGCCTCCATCCATGTTCTCTGATTCCTCCGTTAACGATCCT 780

BoaMYB51.2(Chinese kale)  TTAATGTACCGTAGTGTTGATAATATCGGATTCCCAGGGTTTCTGAATGAGCAACATGTC 840
Bol030761(BRAD)           TTAATGTACCGTAGTGTTGATAATATCGGATTCCCAGGGTTTCTGAATGAGCAACATGTC 840

BoaMYB51.2(Chinese kale)  ATGATGTTGGAAGAGTCTTGTGTTGAGAACACTAAGTTCATGAATGAACTTACGAGGTTT 900
Bol030761(BRAD)           ATGATGTTGGAAGAGTCTTGTGTTGAGAACACTAAGTTCATGAATGAACTTATGAGGTTT 900

BoaMYB51.2(Chinese kale)  CTTCAGGAGGATGTGAACAATGACGTCGAGGTGACGCCGGTCTATGAATATCAAGACGAT 960
Bol030761(BRAD)           CTTCAGGAGGATGTGAACAATGACGTCGAGGTGACGCCGGTCTATGAATATCAAGACGAT 960

BoaMYB51.2(Chinese kale)  TTCGAAGAGATTGGTAACTATTTTGCATGA 990
Bol030761(BRAD)           TTCGAAGAGATTGGTAACTATTTTGCATGA 990
```

(B)

```
BoaMYB51.2(Chinese kale)  MVRTPCCKPELGLKKGAWTPEEDQKLISYLNNHGEGGWRTLPEKAGLKRCGKSCRLRWAN 60
Bol013207(BRAD)           MVRTPCCKPELGLKKGAWTPEEDQKLISYLNNHGEGGWRTLPEKAGLKRCGKSCRLRWAN 60

BoaMYB51.2(Chinese kale)  YLRPDIKRGEFTEDEELSIISLHALHGNKWSDIARGLPGRTDNEIKNYWNTHIKKRLIKK 120
Bol013207(BRAD)           YLRPDIKRGEFTEDEELSIISLHALHGNKWSDIARGLPGRTDNEIKNYWNTHIKKRLIKK 120

BoaMYB51.2(Chinese kale)  GVDPVTHKSLISETSKSENLPEILNNQNIIQTTITSNDDLGNNEKVKNEDKKSRLSSARF 180
Bol013207(BRAD)           GVDPVTHKSLISETSKSENLPEILNNQNIIQTTISSNDDLGNNEKVKNEDKKSRLSSARF 180

BoaMYB51.2(Chinese kale)  LNRVANRFGKKINRSVLSEIIGSGGPLTTTTSVTVDSISDKSMNFSFTPTSYPLNQMTVN 240
Bol013207(BRAD)           LNKVANRVGKKINQSVLSEIIGSGGPLTTTTSVTVDSISDKSMNFSFTPTSYPLNQMTVN 240

BoaMYB51.2(Chinese kale)  GNGNATSPPSMFSDSSVNDPLMYRSVDNIGFPGFLNEQHVMMLEESCVENTKFMNELTRF 300
Bol013207(BRAD)           DNGNATSPPSMFSDSSVNDPLMYRSVDNIGFPGFLNEQHVMMLEESCVENTKFMNELMRF 300

BoaMYB51.2(Chinese kale)  LQEDVNNDVEVTPVYEYQDDFEEIGNYFA 329
Bol013207(BRAD)           LQEDVNNDVEVTPVYEYQDDFEEIGNYFA 329
```

Supplementary Figure S3

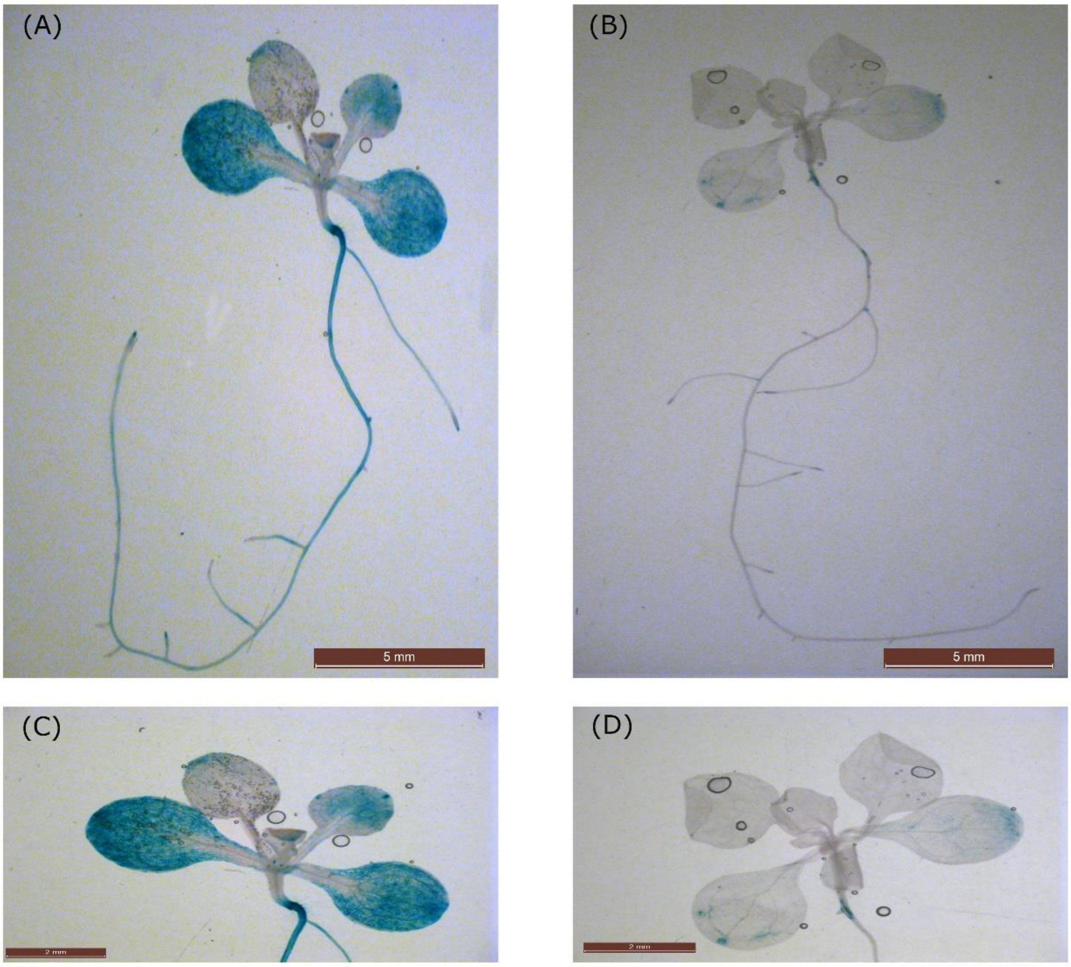

Supplementary Figure S4

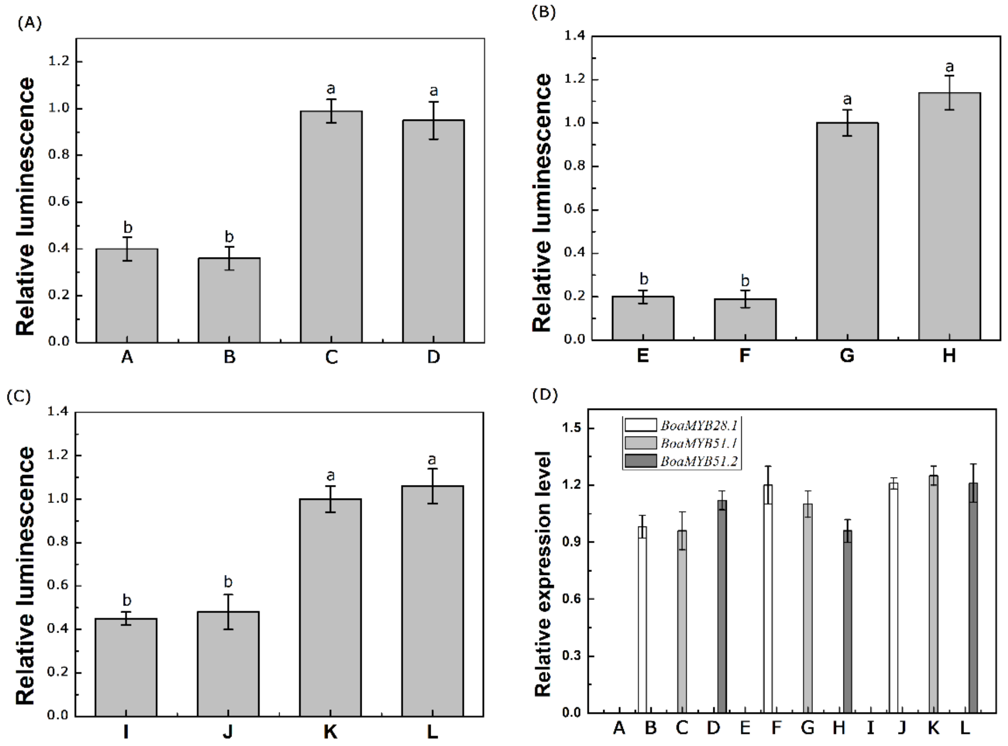

Supplementary Figure S5

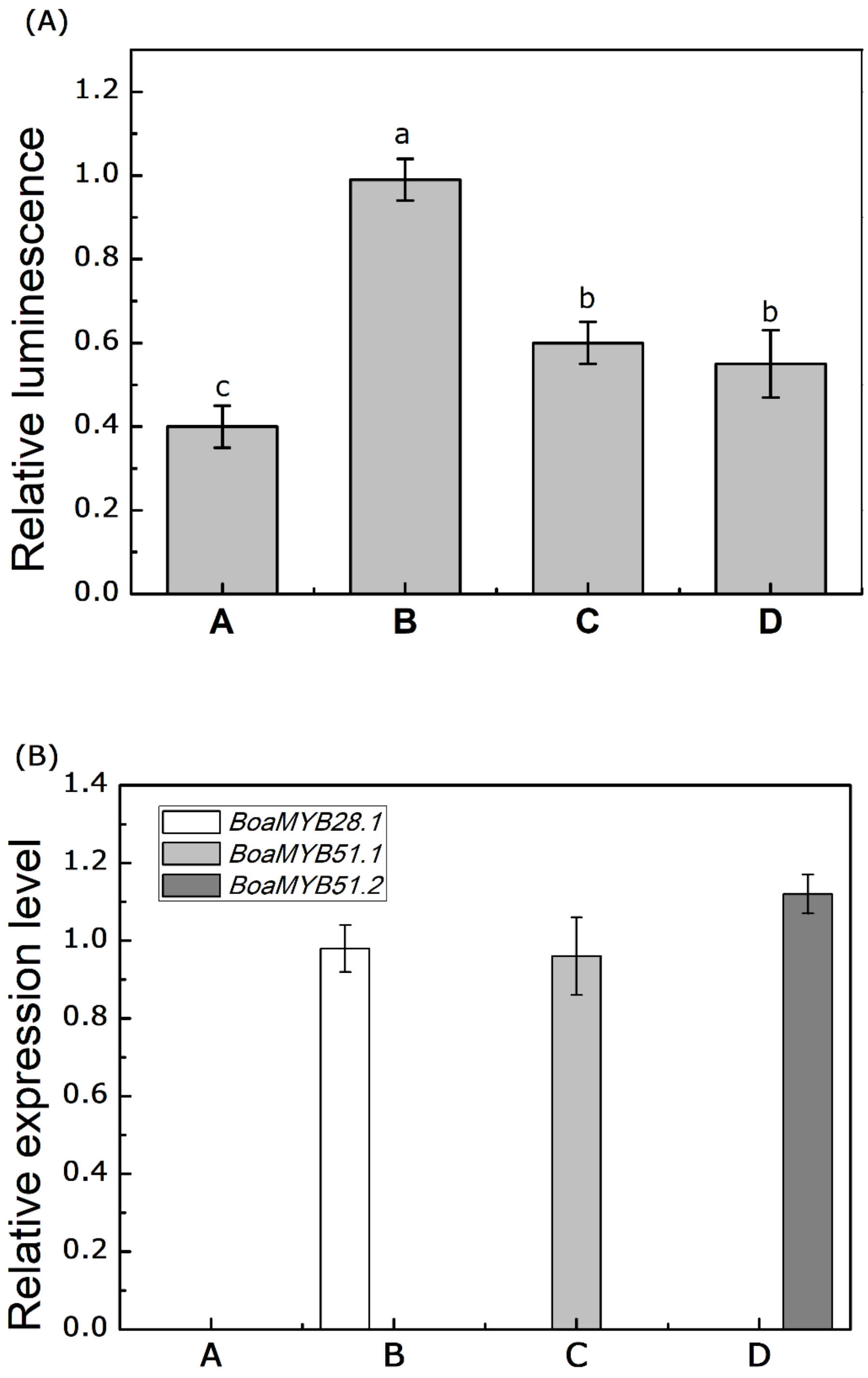

Supplementary Figure S6

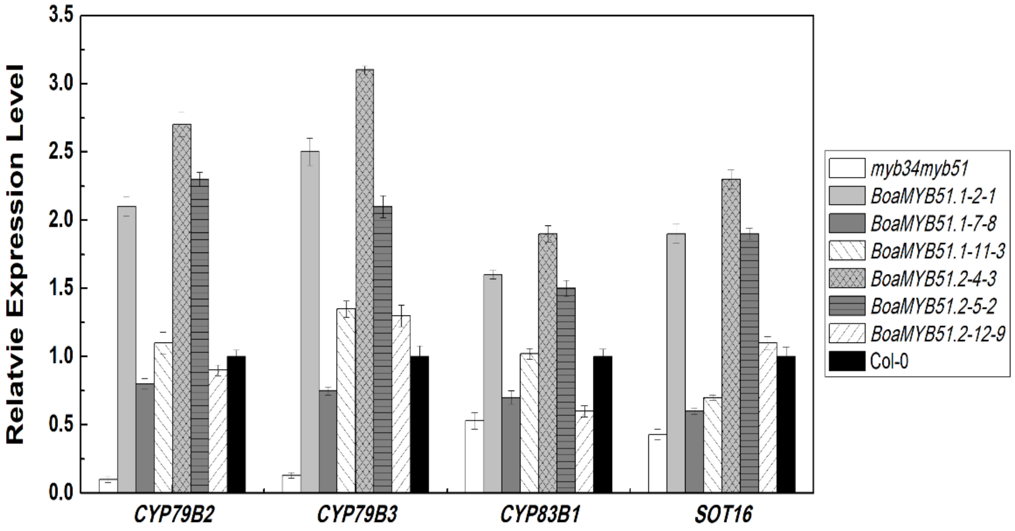

Supplementary Figure S7

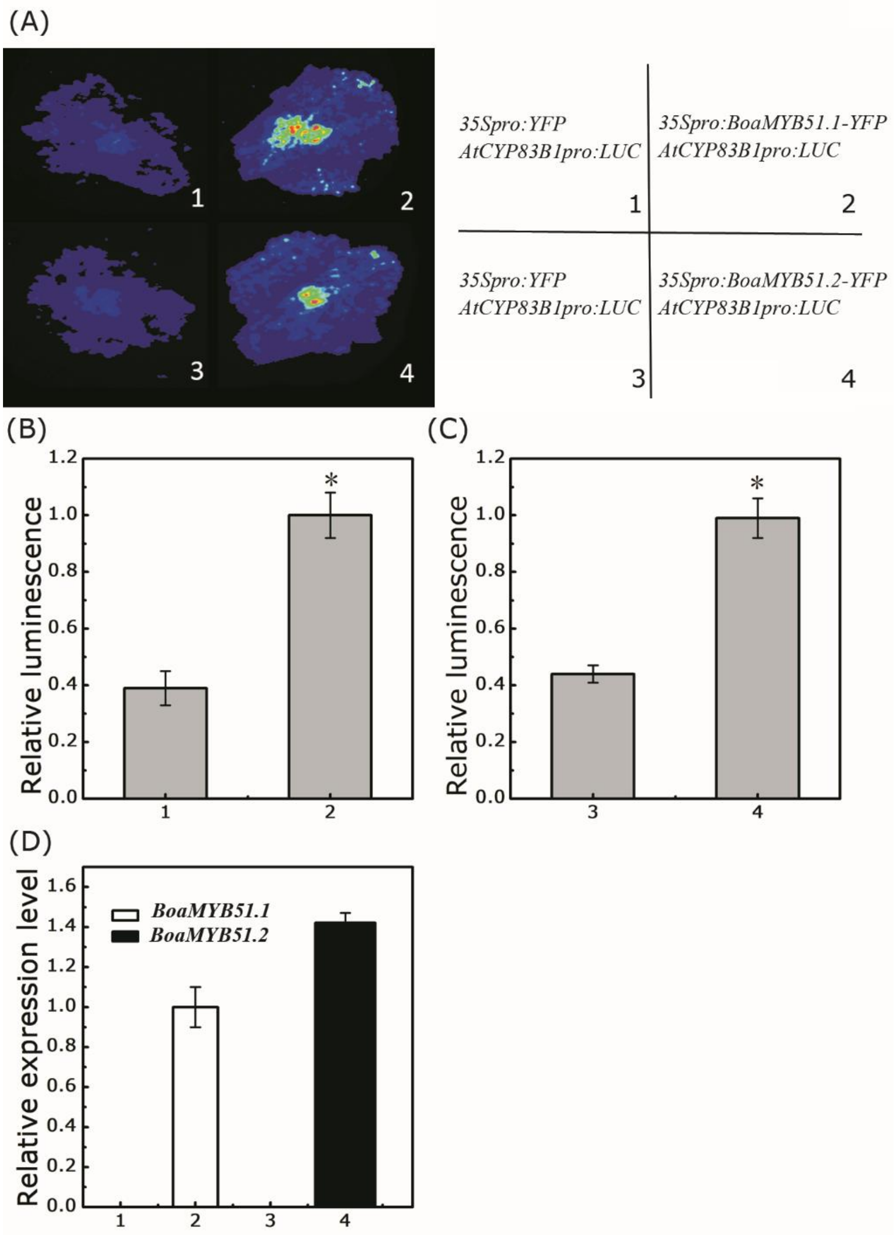

Supplementary Figure S8

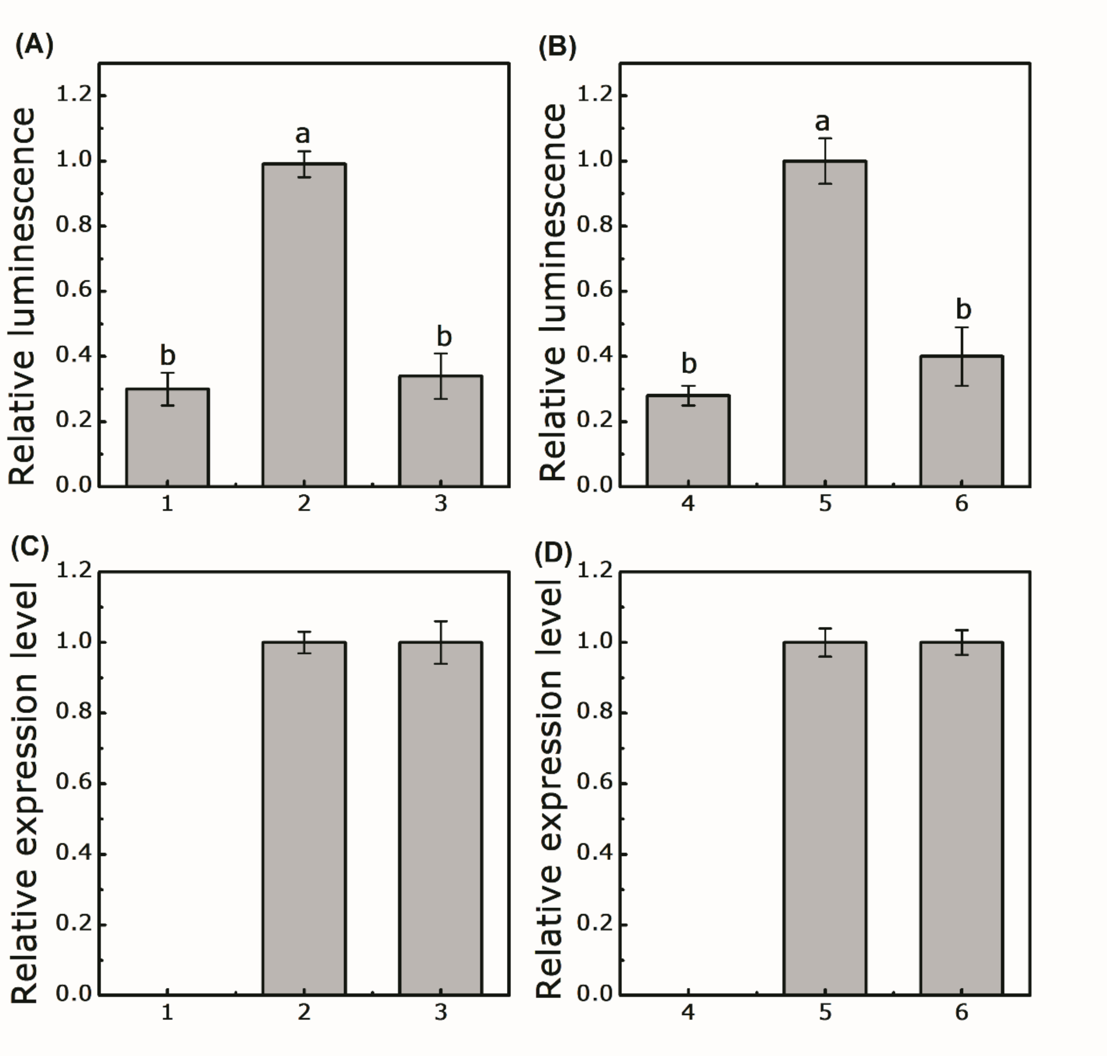

Supplementary Figure S9

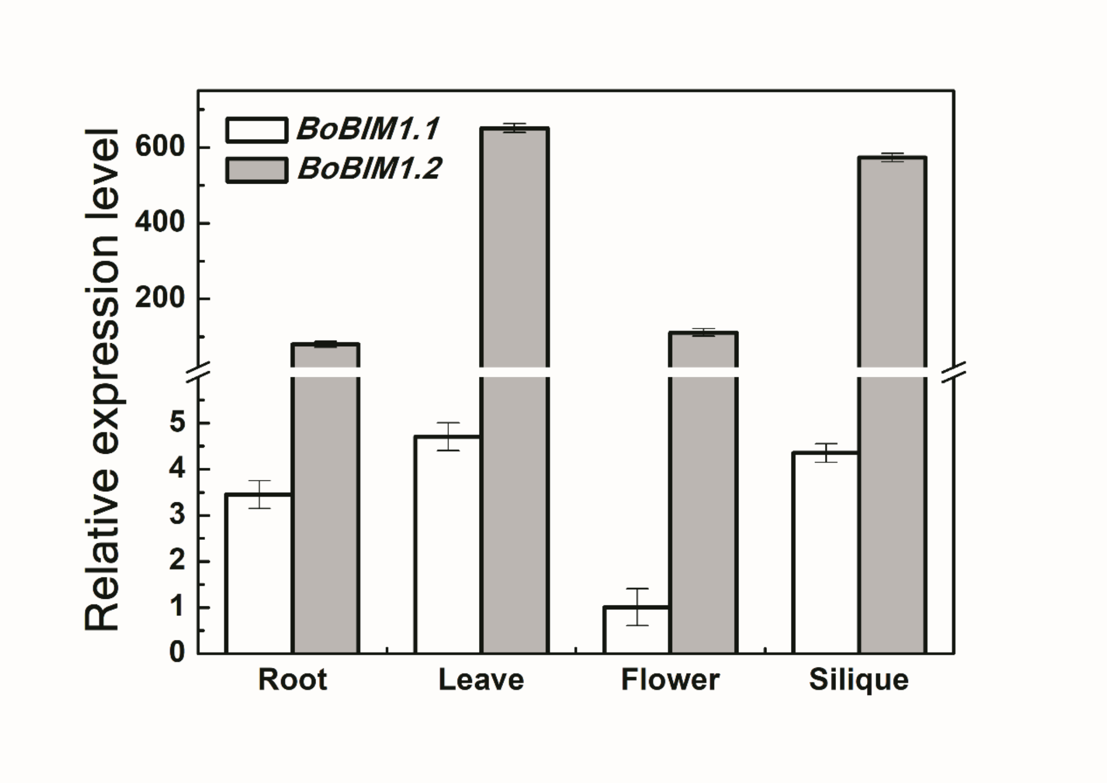

Supplementary Figure S10

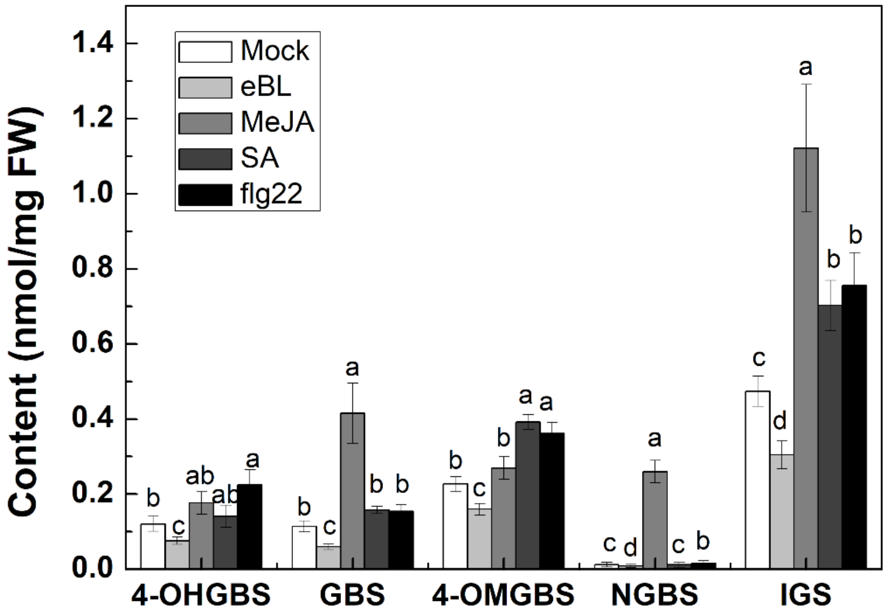

Supplementary Figure S11

```
BoSOT16.1pro  TTTTC--ATCAGAAAATAAAGTGTTGACGGGTTTGTTTGTTTGGAAGCTGCATAGAAAAT  803
              |||||  || || |||||||||||  | |||       | ||||||||||||  |||| |
AtSOT16pro    TTTTCTAATTAGTAAATAAAGTGTACAAGGG-------GCTTGGAAGCTGCAGGGAAA-T  684

BoSOT16.1pro  TCAGCACCAGCCTAGAGATAAACGTGGACCGAACTCAGTTAACG-AACGAGACCAACCAA  862
              |||||| | ||||||||||||||||||||||||| ||  ||||| | | | |||||||||
AtSOT16pro    TCAGCAACGGCCTAGAGATAAACGTGGACCGAACACA-ATAACGTAGCCAAACCAACCAA  743

BoSOT16.1pro  ACCTCTACCTAC-----AAAGTCCATTTCTAAAACGTGACCTTTTAGCTGCAACAACAAC  917
               |||||  ||||     ||  ||||||||||||||||||  ||||    | | |||||||
AtSOT16pro    GCCTCTCTCTACCTGCTAATTTCCATTTCTAAAACGTGA--TTTTTTTAGTACCAACAAC  801

BoSOT16.1pro  CACTTCTCCTC  928
              ||||||| |||
AtSOT16pro    CACTTCTTCTC  812
```

Supplementary Figure S12

(A)

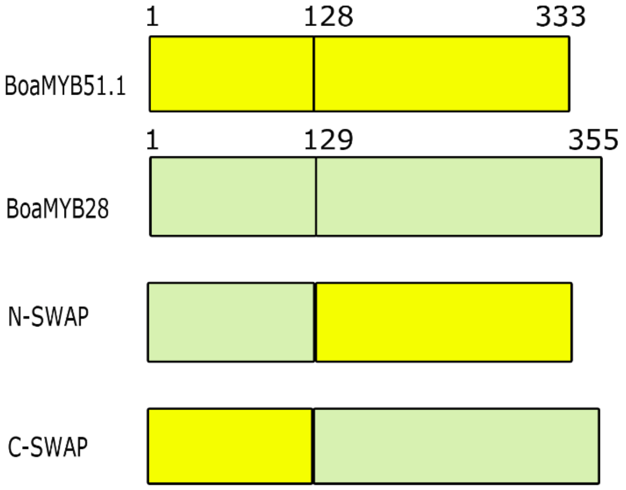

(B)

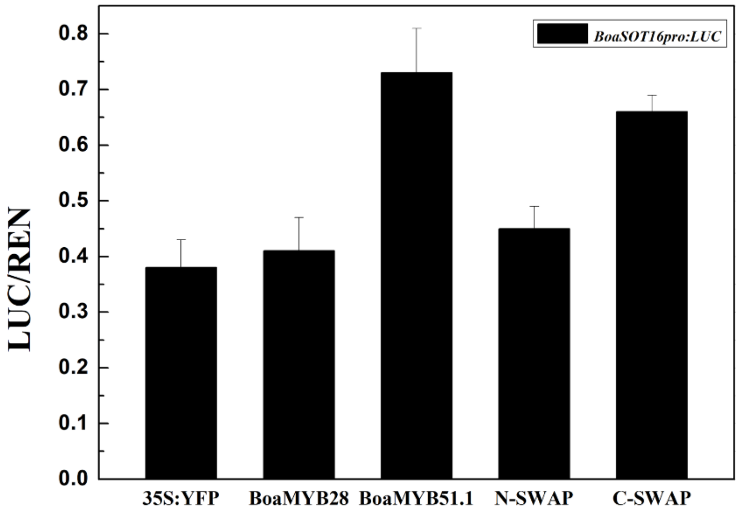

(C)

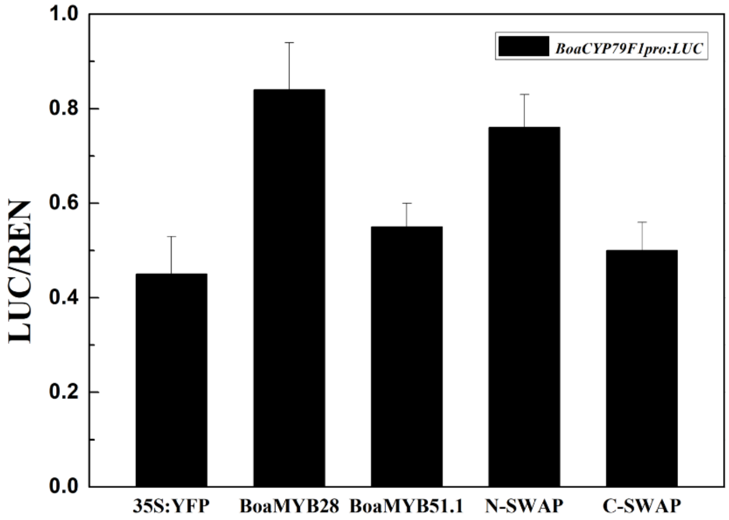

Supplementary Figure S13

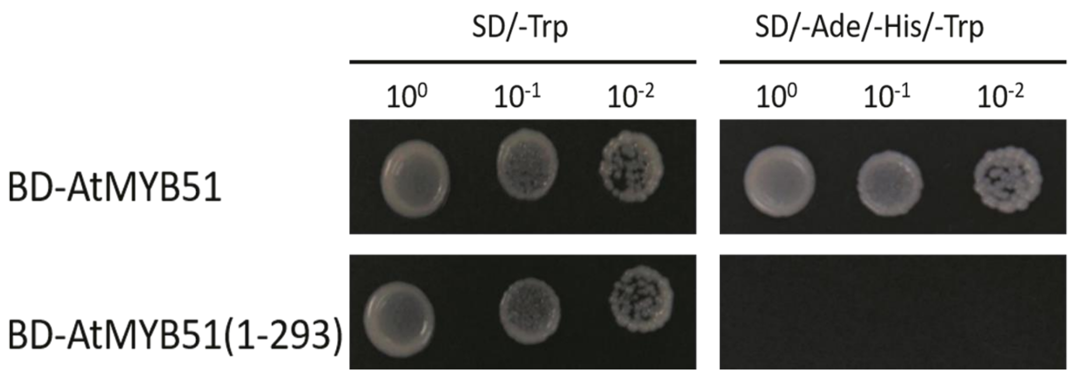

Supplementary Figure S14

(A)

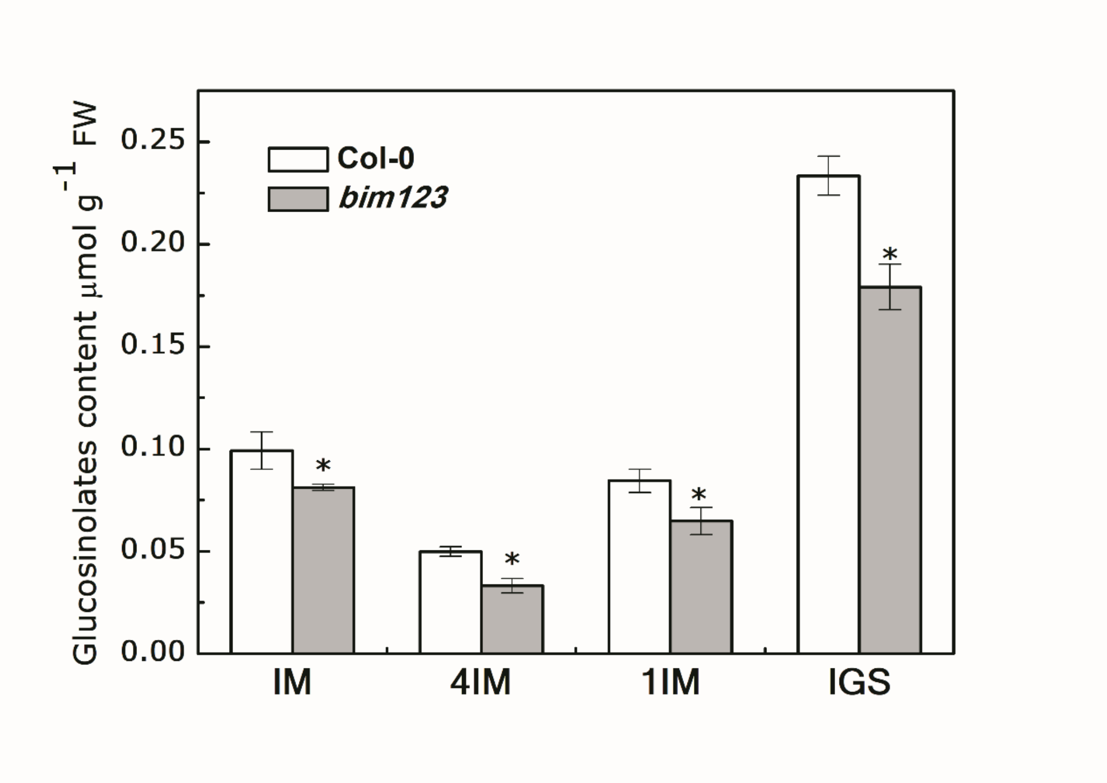

(B)

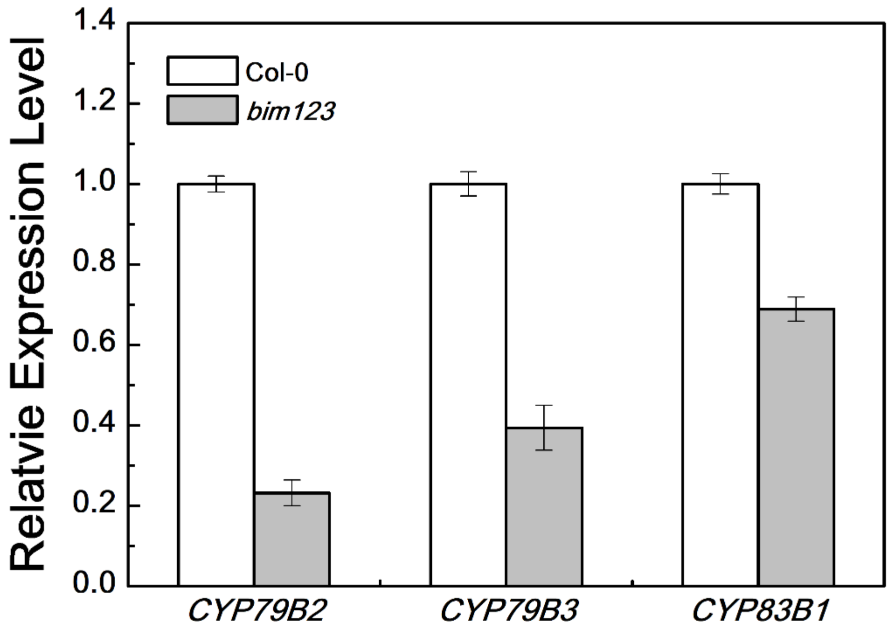

Supplement: Supplementary file 1 [file Data_Sheet_1.PDF]
